# Silk garments plus standard care compared with standard care for treating eczema in children: A randomised, controlled, observer-blind, pragmatic trial (CLOTHES Trial)

**DOI:** 10.1371/journal.pmed.1002280

**Published:** 2017-04-11

**Authors:** Kim S. Thomas, Lucy E. Bradshaw, Tracey H. Sach, Jonathan M. Batchelor, Sandra Lawton, Eleanor F. Harrison, Rachel H. Haines, Amina Ahmed, Hywel C. Williams, Taraneh Dean, Nigel P. Burrows, Ian Pollock, Joanne Llewellyn, Clare Crang, Jane D. Grundy, Juliet Guiness, Andrew Gribbin, Eleanor J. Mitchell, Fiona Cowdell, Sara J Brown, Alan A. Montgomery, UK Dermatology Clinical Trials Network’s CLOTHES Trial Team

**Affiliations:** 1 Centre of Evidence Based Dermatology, University of Nottingham, Nottingham, United Kingdom; 2 Nottingham Clinical Trials Unit, University of Nottingham, Nottingham, United Kingdom; 3 Health Economics Group, Norwich Medical School, University of East Anglia, Norwich, United Kingdom; 4 Nottingham University Hospitals NHS Trust, Queens Medical Centre, Nottingham, United Kingdom; 5 Patient and Public Involvement Representative, Nottingham, United Kingdom; 6 Faculty of Science, University of Portsmouth, Portsmouth, United Kingdom; 7 University of Brighton, Brighton, United Kingdom; 8 Cambridge University Hospitals NHS Foundation Trust, Addenbrooke’s Hospital, Cambridge, United Kingdom; 9 Royal Free London NHS Foundation Trust, Barnet Hospital, Barnet, United Kingdom; 10 Isle of Wight NHS Trust, St. Mary’s Hospital, Newport, United Kingdom; 11 Portsmouth Hospitals NHS Trust, Queen Alexandra Hospital, Portsmouth, United Kingdom; 12 Faculty of Health Education and Life Sciences, Birmingham City University, Birmingham, United Kingdom; 13 Skin Research Group, University of Dundee, Dundee, United Kingdom; 14 Department of Dermatology, Ninewells Hospital and Medical School, Dundee, United Kingdom; Makerere University Medical School, UGANDA

## Abstract

**Background:**

The role of clothing in the management of eczema (also called atopic dermatitis or atopic eczema) is poorly understood. This trial evaluated the effectiveness and cost-effectiveness of silk garments (in addition to standard care) for the management of eczema in children with moderate to severe disease.

**Methods and findings:**

This was a parallel-group, randomised, controlled, observer-blind trial. Children aged 1 to 15 y with moderate to severe eczema were recruited from secondary care and the community at five UK medical centres. Participants were allocated using online randomisation (1:1) to standard care or to standard care plus silk garments, stratified by age and recruiting centre. Silk garments were worn for 6 mo. Primary outcome (eczema severity) was assessed at baseline, 2, 4, and 6 mo, by nurses blinded to treatment allocation, using the Eczema Area and Severity Index (EASI), which was log-transformed for analysis (intention-to-treat analysis). A safety outcome was number of skin infections.

Three hundred children were randomised (26 November 2013 to 5 May 2015): 42% girls, 79% white, mean age 5 y. Primary analysis included 282/300 (94%) children (*n* = 141 in each group). The garments were worn more often at night than in the day (median of 81% of nights [25th to 75th centile 57% to 96%] and 34% of days [25th to 75th centile 10% to 76%]). Geometric mean EASI scores at baseline, 2, 4, and 6 mo were, respectively, 9.2, 6.4, 5.8, and 5.4 for silk clothing and 8.4, 6.6, 6.0, and 5.4 for standard care. There was no evidence of any difference between the groups in EASI score averaged over all follow-up visits adjusted for baseline EASI score, age, and centre: adjusted ratio of geometric means 0.95, 95% CI 0.85 to 1.07, (p = 0.43). This confidence interval is equivalent to a difference of −1.5 to 0.5 in the original EASI units, which is not clinically important. Skin infections occurred in 36/142 (25%) and 39/141 (28%) of children in the silk clothing and standard care groups, respectively. Even if the small observed treatment effect was genuine, the incremental cost per quality-adjusted life year was £56,811 in the base case analysis from a National Health Service perspective, suggesting that silk garments are unlikely to be cost-effective using currently accepted thresholds. The main limitation of the study is that use of an objective primary outcome, whilst minimising detection bias, may have underestimated treatment effects.

**Conclusions:**

Silk clothing is unlikely to provide additional benefit over standard care in children with moderate to severe eczema.

**Trial registration:**

Current Controlled Trials ISRCTN77261365

## Abstract 2

In a randomized controlled trial, Kim Suzanne Thomas and colleagues examine the effectiveness of silk garments vs. standard care for treating eczema in children.

## Author summary

**Why was this study done?:**

- Prior to this trial, evidence on the use of silk garments for the management of eczema was limited.
- Three randomised controlled trials (RCTs) had been conducted, but these were small (74 participants in total) and at risk of bias.
- The existing evidence was insufficient to guide clinical practice on the use of silk clothing in the management of eczema, and no cost-effectiveness analyses had been undertaken.

**What did the researchers do and find?:**

- We conducted a pragmatic, observer-blind RCT that recruited 300 children with moderate to severe eczema and followed them for six months.
- Participants were randomised to receive standard eczema care plus silk clothing (100% sericin-free silk garments; DermaSilk or DreamSkin) or standard care alone.
- After six months, there was no evidence of a difference between the groups in eczema severity (Eczema Area and Severity Index score) assessed by research nurses; the 95% confidence interval ranged from 1.5 points favouring silk clothing to 0.5 points favouring standard care, which is not a clinically important difference.
- Even if the potential small benefit of silk garments was genuine, our analysis suggests that they are unlikely to be cost-effective within currently accepted thresholds, with an incremental cost per quality-adjusted life year of £56,811.

**What do these findings mean?:**

- The CLOTHES Trial is the first large, independent RCT to have evaluated silk garments for the management of eczema.
- The results of this trial suggest that silk garments are unlikely to provide additional clinical or economic benefits over standard care for children with moderate to severe eczema.
- These results provide robust evidence for health commissioners and prescribers to make informed clinical decisions.

## Introduction

Eczema (also called atopic dermatitis or atopic eczema) is a chronic, itchy inflammatory skin condition that is common throughout the world [1]. Childhood eczema has a substantial impact on the quality of life of children and their families [2]. Many families are keen to identify new ways of managing the symptoms of eczema using non-pharmacological approaches [3].

Clothing may play a role in either soothing or exacerbating eczema symptoms, and patients are commonly advised to avoid wool because of its tendency to worsen itch, and to use cotton or fine weave materials next to the skin [4]. Specialist clothing is now available on prescription in a variety of forms including sericin-free silk, viscose, and silver-impregnated fabrics. These garments are claimed to be beneficial for the management of eczema as they can help to regulate the humidity and temperature of the surface of the skin, are smooth in texture, and may reduce skin damage from scratching. Some products have anti-microbial properties that could help to reduce the bacterial load on the skin, which may be important in eczema [5].

To date, there have been just three small randomised controlled trials (RCTs) of silk clothing for the management of eczema [6–8]. These trials involved very few participants (*n =* 22, 30, and 22 participants, respectively), were of generally short duration, did not incorporate an economic evaluation, and were at risk of bias [9].

In view of the limited evidence for the use of silk clothing for eczema management, the UK National Institute for Health Research Health Technology Assessment programme commissioned the CLOTHing for the relief of Eczema Symptoms (CLOTHES) Trial. The trial had two main objectives: (1) to assess whether use of silk garments plus standard eczema treatment reduces eczema severity in children with moderate to severe eczema compared with standard treatment alone, and (2) if so, to establish the likely cost-effectiveness of silk garments.

## Methods

The protocol for this study has been published [10], and the protocol (S1 Protocol) and statistical analysis plan are available (http://www.nottingham.ac.uk/CLOTHES). The study was approved by the Health Research Authority East Midlands–Nottingham 1 Research Ethics Committee (13/EM/0255), and parents/guardians gave written informed consent (children gave assent as appropriate). The trial was registered on Current Controlled Trials prior to start of recruitment (ISRCTN77261365; 11 October 2013). This study is reported as per CONSORT guidelines (S1 Checklist). A full trial report is available [11].

### Study design

The CLOTHES Trial was a multi-centre, parallel-group, observer-blind, pragmatic RCT with 6 mo of follow-up. Children aged 1 to 15 y were randomised (1:1) to receive silk garments plus standard eczema care or standard eczema care alone. The primary outcome was assessed by research nurses blinded to the treatment allocation at baseline, 2, 4, and 6 mo. The trial included a nested qualitative evaluation and health economic analysis. Changes to the protocol after start of participant recruitment included amendment of the number of *FLG* mutations to be included in the genetic analysis and addition of details of the nested qualitative evaluation.

### Recruitment

Recruitment took place at five UK medical centres: Nottingham University Hospitals NHS Trust, Royal Free London NHS Foundation Trust, Cambridge University Hospitals NHS Foundation Trust, Portsmouth Hospitals NHS Trust, and Isle of Wight NHS Trust. Participants were identified through secondary care, through primary care, or in response to local media advertising.

Children aged 1 to 15 y were enrolled. All had a diagnosis of eczema according to the UK Working Party’s Diagnostic Criteria for Atopic Dermatitis [12] and a score of nine or more on the Nottingham Eczema Severity Score, denoting moderate to severe eczema over the last 12 mo [13]. All participants had at least one area of active eczema on part of the body that would be covered by the garments.

Children were excluded if they had taken systemic medication (e.g., ciclosporin or oral corticosteroids) or had received light therapy for eczema in the preceding 3 mo, had used wet/dry wraps ≥5 times in the last month, had started a new medication or treatment regimen that may affect eczema in the last month, were currently using silk clothing for their eczema and were unwilling to stop during the trial, or were currently taking part in another clinical trial. Only one child was enrolled per family.

### Interventions

The silk garments used in the trial (DermaSilk or DreamSkin) are licensed as a medical device with a CE mark for use in eczema, denoting that they comply with EU legislation and safety requirements. Two brands were included to improve the generalisability of the trial findings, to avoid commercial advantage to any one company, and to limit the financial commitment for the companies that donated the garments.

The garments are made with antimicrobially protected, knitted, sericin-free silk (100%). Sericin is removed from the silk fibres during manufacture because it is a protein that coats the outside of silk fibres and has the potential to cause allergic reactions. Participants received three sets of garments (long-sleeved undershirts and leggings or bodysuits and leggings, depending on the age of the child) and were instructed to wear the clothing as often as possible during the day and at night.

Standardised usage instructions were provided, and participants were advised to allow topical medications to absorb into the skin prior to wearing the garments. Replacement garments were provided if they were worn out, lost, or no longer fitted during the 6-mo period of the trial.

Participants in both the intervention and control group continued with their standard eczema care in line with National Institute for Health and Care Excellence (NICE) guidance [14], including regular emollient use and topical corticosteroids (or calcineurin inhibitors) for controlling inflammation. Participants were asked not to change their standard eczema treatment for the duration of the trial unless medically warranted. If a skin infection was suspected, participants were advised to contact their normal medical team for confirmation of diagnosis and subsequent treatment.

### Outcomes

Core outcomes as defined by the Harmonising Outcomes Measures for Eczema (HOME) initiative [15,16] were included.

### Primary outcome

Eczema severity captured using the Eczema Area and Severity Index (EASI) [17] was assessed by trained research nurses at baseline, 2, 4, and 6 mo. Baseline EASI score was used as a covariate in the analysis model. EASI is a validated scale recommended as the core outcome instrument for eczema signs [18]. EASI scoring involves an evaluation of four eczema signs (erythema [redness], excoriation [scratching], oedema/papulation [swelling and fluid in the skin], and lichenification [thickening of the skin]) and an assessment of percentage area affected by eczema in four body regions (head and neck, upper limbs, trunk, and lower limbs). Higher scores represent more severe disease.

### Secondary outcomes

Secondary outcomes were the following:

1. Global assessment of eczema by research nurses (Investigator Global Assessment [IGA]) [19] and by participants (participant global assessment [PGA]) at baseline, 2, 4, and 6 mo, using a six-point scale (clear, almost clear, mild, moderate, severe, very severe).
2. Self-reported eczema symptoms using the HOME-recommended core outcome instrument [15], the Patient Oriented Eczema Measure (POEM), which captures frequency of itch, sleep loss, bleeding, weeping/oozing, cracking, flaking, and dryness [20]. Higher scores represent more severe disease. POEM scores were collected weekly using an online questionnaire for 6 mo.
3. Three Item Severity (TIS) score [21] at baseline, 2, 4, and 6 mo, assessed by the research nurses at a representative body site (defined as the most bothersome patch of eczema that was covered by the garments).
4. Use of eczema treatments: number of days of use of topical steroids, topical calcineurin inhibitors, emollients, and wet/dry wrapping was assessed weekly using an online questionnaire. Research nurses assessed change in eczema treatment regimen at each visit and categorised it as no change, neutral change, reduction, or escalation.
5. Health-related quality of life at baseline and at 6 mo from the perspectives of the family (Dermatitis Family Impact [DFI]) [22], the main carer (EuroQol EQ-5D-3L) [23], and the child (Atopic Dermatitis Quality of Life [ADQoL] preference-based index [24]; Child Health Utility 9 Dimensions [CHU-9D] [25] in those aged 5 y and over).
6. Durability of the garments and acceptability of use (at 6 mo) and adherence (number of days/nights garments worn, assessed weekly).
7. Within-trial cost-effectiveness from a National Health Service (NHS) perspective using the ADQoL to estimate quality-adjusted life years (QALYs). ADQoL is a preference-based utility instrument with four eczema-specific domains covering ability to join in activities, mood, ability to be comforted, and sleep loss. The resulting 16 possible health states range in utility from 0.356 (worst state) to 0.841 (best state) [24].

### Safety outcomes

Safety outcomes were skin infections requiring antibiotic or antiviral treatment and serious adverse events (SAEs) related to eczema.

### Sample size

Three hundred participants provided 90% power at the 5% significance level (two-tailed) to detect a difference of three points between the groups in mean EASI score. Although this between-group difference is approximately half the published minimum clinically important difference for EASI (suggested from one study in adults receiving systemic therapy) [26], we wanted to be sure that a clinically important difference was not missed. Sample size was based on repeated measures analysis of covariance, a standard deviation (SD) of 13, a correlation between EASI scores at different time points of 0.6, and a loss to follow-up of 10%.

### Randomisation and blinding

Randomisation was stratified by recruiting centre and by participant age: <2 y, 2 to 5 y, and >5 y. A computer-generated pseudo-random code with random permuted blocks of randomly varying size was created by the Nottingham Clinical Trials Unit. Research nurses accessed the randomisation website via unique user logins. The sequence of treatment allocations remained concealed until the database was locked at the end of the study, when it was revealed to data analysts.

Staff at the coordinating centre sent confirmation of treatment allocation to participants (along with the silk clothing as necessary). Whilst it was not possible to blind participants to their treatment allocation, efforts were made to minimise expectation bias by emphasising in the trial documents that the evidence supporting the use of silk garments for eczema was limited and that it was not yet known if such clothing offered any benefit over standard care. Participant-facing study documents also avoided the use of value-laden terms such as “specialist” or “therapeutic” clothing.

In order to preserve blinding of the research nurses, participants were reminded in the study literature and in their clinic appointment letters/texts not to wear the clothing when they attended clinic or to mention the clothing when talking to the research nurses. All questions relating to the acceptability and use of the clothing were completed using either postal or online questionnaires, and telephone and email contact with participants was made by staff from the coordinating centre whenever possible. If the research nurses became unblinded, this was recorded.

### *FLG* genotype analysis

Saliva samples were collected for DNA extraction and *FLG* genotyping. Only participants of white European ethnicity were included in this analysis, because *FLG* mutations are ethnically specific. Results for the four most prevalent loss-of-function mutations in the white European population (R501X, 2282del4, R2447X, and S3247X) were obtained for 217 individuals and were used to define genotype categories: *FLG* wild type (no mutations identified), *FLG* heterozygote (one *FLG* null mutation), and *FLG* homozygote or compound heterozygote (two *FLG* null mutations).

### Statistical methods

Analyses were carried out by L. E. B. (trial statistician) using Stata/SE 13.1. The main approach to analysis was modified intention to treat, i.e., analysis according to randomised group regardless of adherence to allocation and including participants who provided data for at least one follow-up time point. Estimates of the intervention effect are presented with 95% confidence intervals and *p*-values. All regression models included the randomisation stratification variables (recruiting centre and age) as covariates, and baseline scores, if measured. Adjusted differences in means for the intervention group compared to the standard care group are presented for continuous outcomes, and adjusted risk differences and relative risks for binary outcomes. For outcomes collected at the 2-, 4-, and 6-mo visits, we explored whether the effect of the trial garments on the outcome changed over the study period by including an interaction term between treatment group and time point in the model. As there was no evidence of a differential effect over time for any outcomes, we report a single estimate per outcome that averages the treatment effect over all time points.

The primary analysis used a multilevel model with observations at 2, 4, and 6 mo nested within participants. The model used a random intercept and slope at the participant level with an unstructured covariance matrix for these random effects. The model assumed that missing EASI scores were missing at random given the observed data. EASI scores were right skewed at all time points. Diagnostic plots indicated that the assumptions for the multilevel model in the original EASI units were not met. The data were log-transformed for analysis and the treatment effect presented as a ratio of geometric means [27,28]. This ratio was back-transformed to the original EASI units to facilitate interpretation of findings.

Sensitivity analyses for the primary outcome adjusted for variables that had an observed imbalance between the groups at baseline, used multiple imputation for missing outcome data, and explored the impact of adherence in wearing the clothing by estimating the complier average causal effect (CACE) at 6 mo using instrumental variable regression.

A planned subgroup analysis based on presence or absence of loss-of-function mutations in *FLG* (which are associated with impaired skin barrier function and more severe disease) was conducted for the primary outcome by adding an interaction term between allocated treatment and *FLG* genotype (none, one, or two *FLG* null mutations) to the primary analysis model.

The global assessment scores (IGA and PGA) were dichotomized into clear, almost clear, or mild eczema versus moderate, severe, or very severe eczema, and analysed using generalised estimating equations. The mean weekly POEM scores, percentage of days that topical steroids were used, and quality of life outcomes were analysed using linear models (weighted according to the number of questionnaires completed for the weekly POEM and topical steroid use). The TIS score was analysed using the multilevel model framework as outlined above for the primary outcome (not transformed). Changes to treatment regimen were based on whether a participant had reported treatment escalation over the 6-mo RCT period and were analysed using a generalised linear model. Skin infections were analysed using negative binomial regression. SAEs and durability and acceptability of use of the garments were summarised descriptively.

Adherence in wearing the trial clothing was summarised using the percentage of days and nights that the study clothing was worn. Participants were classified as being broadly adherent if they wore the trial clothing for at least 50% of the days or 50% of the nights. This classification was done for participants for whom at least half (12/24) of the weekly questionnaires were completed, and sensitivity analysis explored the impact of different assumptions for those participants who completed less than 50% of the weekly questionnaires. Adherence with the trial clothing was explored descriptively according to age and baseline eczema severity using correlation coefficients.

Full details of the analysis are documented in the statistical analysis plan, which was finalised prior to database lock and release of treatment allocation codes for analysis.

Following concerns that the baseline EASI scores appeared lower than might be expected for children with moderate to severe eczema, an additional post hoc analysis was conducted to explore the interaction between baseline severity and treatment group by adding an interaction term between allocated group and baseline EASI score (log-transformed and continuous) to the primary analysis model.

### Patient involvement

Public and patient involvement (PPI) was embedded throughout the CLOTHES Trial. Various PPI methods such as online surveys, discussion groups, and patient panels were used to inform multiple aspects of the trial design including choice of comparator, eligibility criteria, potential barriers to participation, and outcome measures. PPI members of the trial team also contributed to the development of patient-facing study materials and took part in media interviews to enhance recruitment. A PPI representative was a co-applicant on the grant and was involved in all stages from trial design through to data interpretation and write up, and another PPI representative was a member of the trial steering committee.

The study results will be published on the CLOTHES Trial website, and a written summary and child-friendly animated film will be sent to trial participants.

### Health economics

The within-trial economic analysis (conducted by T. H. S. using Stata/SE 14.1) compared the costs and QALYs in the standard care and intervention groups from the perspective of the NHS. We attached published unit costs (2014–2015 UK pounds sterling) [29–31] to individual-level quantities of resource use (S1 Table) and estimated the mean cost per participant incorporating the cost of the intervention and wider healthcare resource use (primary care, secondary care, and medications).

QALYs were estimated using linear interpolation and area under the curve analysis, adjusting for baseline values, age, and recruitment centre. A regression-based approach (seemingly unrelated regression equations) [32] was used for the statistical analysis. The level of uncertainty associated with the decision over which option was most cost-effective was explored using non-parametric bootstrapping [33] to construct the cost-effectiveness acceptability curve [34]. Neither costs nor QALYs were discounted reflecting the time frame.

To test the impact of taking an alternative approach to costing the silk garments, sensitivity analysis included an estimate of the amount pharmacists are reimbursed for each item of clothing they prescribe. This analysis was based on the NHS Business Services Authority formula to estimate the actual cost to the NHS. The analysis was rerun using the March 2015 tariff data [35], where the average discount was 7.43% and the pharmacist’s professional fee £0.90 per prescription item.

## Results

### Recruitment and retention

**Fig 1:**
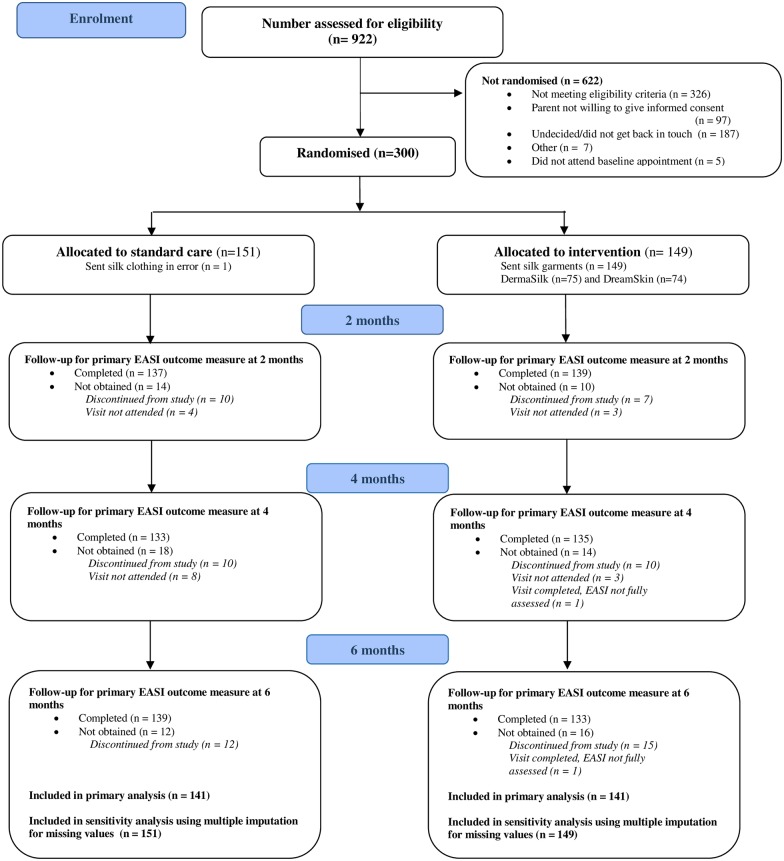
Participant flow diagram. EASI, Eczema Area and Severity Index.

Three hundred children were randomised between 26 November 2013 and 5 May 2015 (last study visit 21 October 2015). The primary analysis included 141 participants in each group who had at least one primary outcome assessment after baseline (Fig 1). For all but four participants, outcome assessments were performed by the same nurse at all study visits.

For the weekly online questionnaires (24 questionnaires over 6 mo), 126/149 (85%) participants in the intervention group and 127/151 (84%) participants in the standard care group completed 12 questionnaires or more. The median number completed was 22 (25th to 75th centile 17 to 24) in both groups.

### Baseline characteristics

Participants had a mean age of 5 y, 42% were girls, and 79% were white. At recruitment, 72% had moderate or severe eczema, as judged by the IGA (Table 1). Demographic and clinical characteristics were well balanced at baseline apart from gender and parental reported history of asthma and food allergy (Table 1). The mean baseline EASI score was slightly higher in the intervention group as more children had a baseline EASI score of over 30 points (14 participants in the intervention group, four participants in the standard care group). However, the median and interquartile range for EASI score were similar between the groups (Table 2).

**Table 1:** Baseline demographic and clinical characteristics.

Characteristic	Standard care (*n* = 151)	Intervention (*n* = 149)	Total (*n* = 300)
***Demographics***			
**Age (years)**			
Mean [SD]	5 [3.6]	5.1 [3.7]	5.1 [3.6]
Median [25th, 75th centile]	4 [2, 8]	4 [2, 7]	4 [2, 7.5]
Minimum, maximum	1, 14	1, 15	1, 15
**Gender**			
Boys	82 (54%)	92 (62%)	174 (58%)
Girls	69 (46%)	57 (38%)	126 (42%)
**Ethnicity**			
White	123 (81%)	114 (77%)	237 (79%)
Indian/Pakistani/Bangladeshi	8 (5%)	7 (5%)	15 (5%)
Black	6 (4%)	6 (4%)	12 (4%)
Chinese	1 (1%)	3 (2%)	4 (1%)
Other Asian (non-Chinese)	0	4 (3%)	4 (1%)
Mixed race	12 (8%)	13 (9%)	25 (8%)
Other	1 (1%)	2 (1%)	3 (1%)
***Clinical characteristics***			
**History of atopy (self-reported)**			
Asthma	57 (38%)	46 (31%)	103 (34%)
Allergic rhinitis	60 (40%)	56 (38%)	116 (39%)
Food allergy	80 (53%)	68 (46%)	148 (49%)
Anaphylaxis	23 (15%)	23 (15%)	46 (15%)
**Type of eczema**			
Discoid	19 (13%)	17 (11%)	36 (12%)
Flexural	144 (95%)	147 (99%)	291 (97%)
**Location of eczema**			
Head and neck	115 (76%)	120 (81%)	235 (78%)
Hands and wrists	116 (77%)	108 (72%)	224 (75%)
Feet and ankles	100 (66%)	96 (64%)	196 (65%)
Limbs	151 (100%)	149 (100%)	300 (100%)
Trunk	128 (85%)	122 (82%)	250 (83%)
**Previous medical care**			
General practitioner only	41 (27%)	40 (27%)	81 (27%)
General practitioner and secondary care	110 (73%)	109 (73%)	219 (73%)
**Medication used in the month prior to randomisation**			
Emollients	150 (99%)	146 (98%)	296 (99%)
Topical steroids	136 (90%)	130 (87%)	266 (89%)
Calcineurin inhibitors	14 (9%)	15 (10%)	29 (10%)
Wet/dry wraps (1–4 times)	13 (9%)	14 (9%)	27 (9%)
Nottingham Eczema Severity Score*			
Moderate eczema in last year (9–11)	28 (19%)	30 (20%)	58 (19%)
Severe eczema in last year (12–15)	123 (81%)	119 (80%)	242 (81%)
**Investigator Global Assessment on day randomised**			
Almost clear	4 (3%)	2 (1%)	6 (2%)
Mild	39 (26%)	39 (26%)	78 (26%)
Moderate	77 (51%)	67 (45%)	144 (48%)
Severe	30 (20%)	36 (24%)	66 (22%)
Very severe	1 (1%)	5 (3%)	6 (2%)
Patient Oriented Eczema Measure, mean [SD]*	16.6 [4.8]	17.3 [5.8]	17 [5.4]
**Healthcare resource use (2014–2105 UK£) (4 wk pre-trial), mean [SD]**	£34.82 [69.14]	£35.60 [69.46]	£35.20 [69.17]
***FLG* genotype**^$^ **(using mutations R501X, 2282del4, R2447X, S3247X) for white individuals only**			
*n*	123	114	237
No mutations	72 (59%)	71 (62%)	143 (60%)
One *FLG* null mutation	31 (25%)	20 (18%)	51 (22%)
Two *FLG* null mutations	12 (10%)	11 (10%)	23 (10%)
Not known	8 (7%)	12 (11%)	20 (8%)

Data are *n* (percent) unless otherwise specified. Categories for history of atopy, type of eczema, and location of eczema are not mutually exclusive.

*Higher values represent more severe AE.

^$^For children for whom informed consent for genetic study was given and the saliva sample was taken. Data are presented for individuals of white European ethnicity only as *FLG* mutations are population-specific and the mutations tested are prevalent in white European individuals.

SD, standard deviation.

**Table 2:** Outcomes assessed during clinic visits.

Outcome and allocated group	Time point				Adjusted intervention effect* (95% CI), *p*-value
	Baseline	2 mo	4 mo	6 mo	
***Blinded outcomes***					
**Eczema Area and Severity Index (primary outcome)**					Ratio of geometric means 0.95 (0.85, 1.07), *p* = 0.43
**Standard care**					
*n*	151	137	133	139	
Median [25th, 75th centile]	7.3 [4.2, 12]	5.3 [2.5, 10.5]	4.3 [2.1, 10]	4.2 [2, 9.2]	
Geometric mean	8.4	6.6	6.0	5.4	
**Intervention**					
*n*	149	139	135	133	
Median [25th, 75th centile]	7 [4.1, 15.4]	4.9 [2.2, 9.9]	4.1 [2.2, 9.4]	4 [1.9, 7.9]	
Geometric mean	9.2	6.4	5.8	5.4	
**Three Item Severity score, mean [SD] (*n*)**					Difference in means 0.09 (−0.22, 0.40), *p* = 0.57
Standard care	4.9 [1.8] (n = 151)	4.0 [1.9] (n = 137)	4.1 [2.2] (n = 133)	3.7 [1.9] (n = 139)	
Intervention	4.9 [1.8] (n = 149)	4.1 [2.0] (n = 139)	4.1 [2.1] (n = 136)	3.7 [2.0] (n = 134)	
**Investigator Global Assessment of moderate, severe, or very severe eczema, *n/N* (percent)**					Risk difference −0.1% (−9.3%, 6.3%), *p* = 0.70; relative risk 0.98 (0.82, 1.12), *p* = 0.63
Standard care	108/151 (72%)	72/137 (53%)	63/133 (47%)	56/139 (40%)	
Intervention	108/149 (72%)	71/139 (51%)	60/136 (44%)	58/134 (43%)	
***Unblinded outcomes***					
**Participant global assessment of moderate, severe, or very severe eczema, *n/N* (percent)**					Risk difference −10.1%, (−18.3%, −2.0%), *p* = 0.01; relative risk 0.83 (0.70, 0.98), *p* = 0.03
Standard care	113/151 (75%)	82/137 (60%)	72/133 (54%)	60/139 (43%)	
Intervention	98/149 (66%)	62/139 (45%)	56/135 (41%)	51/134 (38%)	
**Treatment escalation since previous visit, *n/N* (percent)**					Risk difference (any escalation^$^) −5.3% (−16.3%, 5.7%), *p* = 0.34; relative risk (any escalation^$^) 0.87 (0.62, 1.22), *p* = 0.43
Standard care	—	34/137 (25%)	16/133 (12%)	16/139 (12%)	
Intervention	—	15/139 (11%)	16/136 (12%)	16/134 (12%)	
***Quality of life outcomes***^*#*^					
**DFI questionnaire, mean [SD] (*n*)**					Difference in means −0.8 (−2.1, 0.4), *p* = 0.18
Standard care	12.0 [6.3] (n = 151)	N/A	N/A	8.6 [6.8] (n = 138)	
Intervention	12.4 [6.6] (n = 149)	N/A	N/A	7.6 [6.1] (n = 133)	
**ADQoL, mean [SD] (*n*)**					Difference in means 0.0260 (−0.0018, 0.0539), *p* = 0.07
Standard care	0.6952 [0.1300] (n = 151)	N/A	N/A	0.7292 [0.1308] (n = 139)	
Intervention	0.6883 [0.1409] (n = 149)	N/A	N/A	0.7515 [0.1273] (n = 134)	
**CHU-9D (5 y and over only), mean [SD] (*n*)**					Difference in means −0.0243 (−0.0584, 0.0098), *p* = 0.16
Standard care	0.8292 [0.1263] (n = 64)	N/A	N/A	0.8828 [0.1059] (n = 67)	
Intervention	0.8386 [0.1115] (n = 70)	N/A	N/A	0.8677 [0.1114] (n = 65)	
**EQ-5D-3L parent health-related quality of life, mean [SD] (*n*)**					Difference in means 0.0115 (−0.0185, 0.0415), *p* = 0.45
Standard care	0.8983 [0.1612] (n = 151)	N/A	N/A	0.9107 [0.1529] (n = 138)	
Intervention	0.9018 [0.1710] (n = 147)	N/A	N/A	0.9184 [0.1564] (n = 134)	

*In all, 282 participants were included in the analysis model for the Eczema Area and Severity Index (EASI) (*n* = 141 in each group; note that one participant in the intervention group attended two follow-up visits but EASI was not fully assessed at either of these visits as the child and parent did not want lower limbs assessed); 283 participants were included in the analysis models for the global assessments and Three Item Severity score (*n* = 141 standard care, *n* = 142 intervention); 271 participants were included in the analysis model for DFI (*n* = 138 standard care, *n* = 133 intervention). All analyses were adjusted for recruiting centre, age, and baseline value of the outcome, if it was measured.

^$^Treatment escalation analysed as any treatment escalation between baseline and 6 mo: escalation was reported by 50/140 participants (36%) in the standard care group and 42/138 (30%) in the intervention group. Participants who missed visits were included if they had an escalation at any of the visits they attended or if they attended the 6-mo visit and there was neutral or no change or a reduction in treatment.

^#^Ranges for quality of life scores: DFI, 0 to 30; ADQoL, 0.356 to 0.841; CHU-9D, 0.33 to 1; EQ-5D-3L, −0.594 to 1.

ADQoL, Atopic Dermatitis Quality of Life; CHU-9D, Child Health Utility 9 Dimensions; DFI, Dermatitis Family Impact; N/A, not applicable; SD, standard deviation.

### Adherence, contamination, and blinding

**Fig 2:**
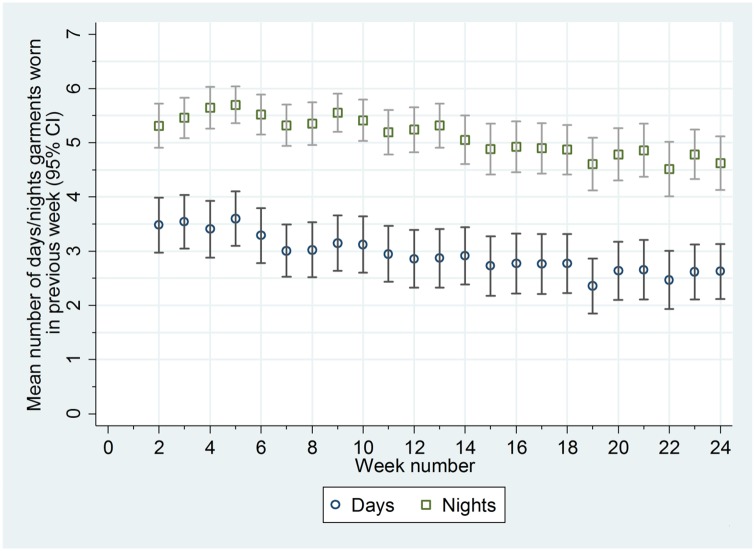
Mean number of days and nights trial garments worn each week.

Adherence in wearing the garments was good. The garments were worn more often at night than in the day (median of 81% of nights [25th to 75th centile 57% to 96%] and 34% of days [25th to 75th centile 10% to 76%]) (Fig 2; S2 Table). Adherence in wearing the garments was not associated with age or eczema severity at baseline (S2 Table). Contamination of the standard care group was low; six participants reported wearing silk clothing during the trial (including one participant who was allocated to the standard care group but was sent the silk clothing in error; this participant was included in the analysis according to randomised allocation).

Acceptability of the garments as assessed at 6 mo suggested that 85/121 (70%) participants were satisfied or very satisfied with the clothing (95% CI 61% to 78%), and 89/121 (74%) participants were either happy or very happy to wear the garments (95% CI 64% to 81%). Some participants raised concerns about the garments, including poor durability and fit.

Research nurses remained blinded to treatment allocation for 289/300 (96%) of participants. Unblinding occurred for three participants in the standard care group and eight in the intervention group.

### Primary outcome

**Fig 3:**
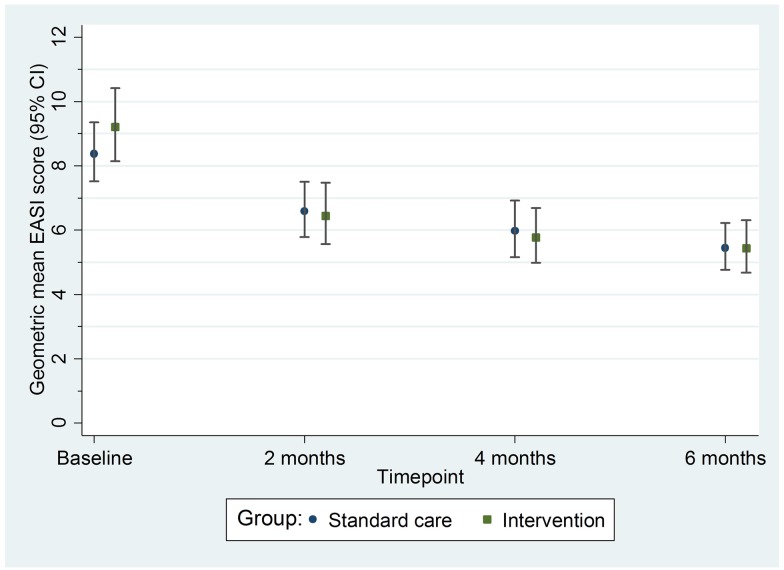
Primary outcome: Geometric mean nurse-assessed eczema severity (EASI score) with 95% confidence intervals. EASI, Eczema Area and Severity Index.

For the primary outcome of eczema severity, there was no difference between the groups in the nurse-assessed EASI scores. For EASI scores averaged over the 2-, 4-, and 6-mo follow-up visits, the adjusted ratio of geometric means was 0.95, with 95% CI 0.85 to 1.07 (*p* = 0.43) (Table 2; Fig 3). This confidence interval equates to a difference of approximately −1.5 to 0.5 points in the original EASI units.

All sensitivity analyses for the primary outcome (adjusting for additional baseline factors, imputing missing values, and exploring the impact of adherence [CACE analysis]) were supportive of the primary analysis (S3 Table). There was no differential effect of the clothing on EASI score (eczema severity) according to *FLG* subgroup (S4 Table) or severity of eczema at baseline (S5 Table).

### Secondary and safety outcomes

**Fig 4:**
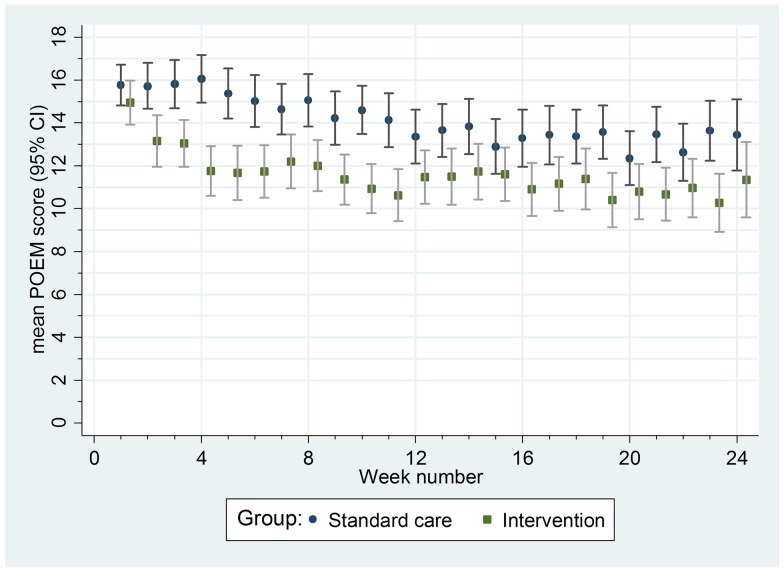
Mean weekly patient-reported symptoms (POEM score) with 95% confidence intervals. POEM, Patient Oriented Eczema Measure.

For the secondary outcomes, there were no between-group differences in nurse-assessed eczema severity (IGA, TIS), quality of life (DFI, EQ-5D-3L, CHU-9D), or medication use (percentage of days eczema medications used, escalation of eczema treatment) (Tables 2 and 3). However, small differences were observed for two of the participant-reported secondary outcomes of eczema severity (PGA, POEM) (Tables 2 and 3; Fig 4). Safety outcomes (number of skin infections and hospitalizations due to eczema) were similar in the two groups (Table 4).

**Table 3:** Secondary outcomes assessed on weekly questionnaires.

Outcome	Standard care (*n* = 147)	Intervention (*n* = 145)	Adjusted difference in means (intervention minus standard care) (95% CI), *p*-value
Participant mean of weekly POEM score during the 6-mo trial*	14.2 [5.5]	11.6 [5.6]	−2.8 (−3.9, −1.8), *p* < 0.001
Percentage of days topical steroids used^$^	44.1 [28.2]	39.3 [27.8]	−3.7 (−9.6, 2.3), *p* = 0.23
Percentage of days emollients used^#^	88.4 [20.1]	86.0 [22.1]	
Percentage of days calcineurin inhibitors used^	5.8 [15.9]	5.7 [16.3]	
Percentage of days wet/dry wraps used^	5.2 [17.1]	3.1 [12.5]	

Data are mean [standard deviation]. Table shows data for participants who completed at least one questionnaire. Summary statistics and analyses reported are weighted according to the number of questionnaires completed.

*Difference in means adjusted for baseline POEM score and stratification variables age and recruiting centre.

^$^Difference in means adjusted for topical steroid use at baseline (yes/no) and stratification variables age and recruiting centre.

^#^Between-group analysis not done: assumptions for model not met because most participants were using emollients most of the time.

^ Between-group analysis not done: assumptions for model not met due to the large number of participants who were not using these treatments.

POEM, Patient Oriented Eczema Measure.

**Table 4:** Safety outcomes.

Outcome	Standard care (*n* = 141)	Intervention (*n* = 142)	Adjusted relative risk (95% CI), *p*-value
Any skin infection during the 6-mo trial, *n* (percent)*	39 (28%)	36 (25%)	0.89 (0.54, 1.47), *p* = 0.66
**Number of skin infections per participant**			
Median [25th, 75th centile]	1 [1, 2]	1 [1, 2]	
Minimum, maximum	1, 5	1, 8	
*n*	39	36	
**Number of inpatient stays per participant due to eczema, *n* (percent)**			
0	139 (99%)	138 (97%)	
1	1 (1%)	2 (1%)	
2	1 (1%)	2 (1%)	
3 or more	0	0	

Table shows data for participants who attended at least one follow-up visit. Percentages for any skin infection and number of inpatient stays use the number of participants attending at least one follow-up visit as the denominator. Skin infections were reported by the parent/main carer and defined as any skin infection that required treatment with antivirals or antibiotics. Inpatient hospital stays for eczema (for any reason) were reported by the parent/main carer.

*Relative risk for skin infections adjusted for stratification variables age and recruiting centre.

### Cost-effectiveness

The economic evaluation included all participants with complete resource use and ADQoL data at baseline and 6 mo (*n =* 273). The cost of a single set of tops and leggings ranged from £66.02 to £155.49, depending on the size of the child. The mean cost of silk garments for 6 mo, including initial and replacement garments, was £318.52 (SD £136.60) per participant in the base case (Table 5). The mean number of sets of garments (tops and leggings) per participant in the base case was 4.15 (SD 1.56). Sixty-one (45.54%) intervention participants received replacement garments over the 6 mo.

**Table 5:** Key findings from the base case economic evaluation.

Outcome	Standard care (*n =* 139), mean (SD)	Intervention (*n =* 134), mean (SD)	Unadjusted (and adjusted) mean difference (intervention minus standard care) (95% CI)
***Health outcomes***			
**Utility (ADQoL)**			
Baseline	0.6959 (0.1288)	0.6879 (0.1418)	−0.0081 (−0.0404, 0.0241)
6 mo	0.7292 (0.1308)	0.7515 (0.1273)	0.0224 (−0.0084, 0.0531)
**QALYs over 6 mo**	0.3563 (0.0562)	0.3598 (0.0561)	0.0036 (−0.0098, 0.0169); adjusted: 0.0064 (−0.0004, 0.0133)
***Costs (2014–2015 UK£)***			
Garments	0.00 (0.00)	318.52 (136.60)	318.52 (295.71, 341.33)
Primary care visits	47.01 (73.71)	36.52 (57.74)	−10.49 (−26.30, 5.33)
Secondary care visits	153.00 (327.13)	213.09 (604.47)	60.09 (−55.16, 175.34)
Prescriptions	120.86 (243.81)	119.82 (244.67)	−1.04 (−59.25, 57.18)
Total healthcare costs, excluding garments	320.86 (446.13)	369.43 (805.88)	48.57 (−105.92, 203.05)
Total healthcare costs, including garments	320.86 (446.13)	687.96 (809.27)	367.09 (212.12, 522.07); adjusted: 364.94 (217.47, 512.42)

Incremental cost-effectiveness ratio = £56,811 per QALY.

ADQoL, Atopic Dermatitis Quality of Life; QALY, quality-adjusted life year; SD, standard deviation.

Combined with wider health resource use, the adjusted mean difference in cost per participant was £364.94 (95% CI £217.47 to £512.42, *p* < 0.001) for those who received silk garments compared to those who did not in the base case (Table 5). The difference in total costs between groups reflects the cost of the intervention; wider NHS costs were not significantly different between groups (£48.57 higher per participant on average in the intervention group, 95% CI −£105.92 to £203.05, *p* = 0.537). For resource use and costs for all resource items, see S6 and S7 Tables.

The adjusted mean difference in QALY per participant was 0.0064 (95% CI −0.0004 to 0.0133, *p* = 0.07) (Table 5). The adjusted incremental cost per QALY was £56,811, suggesting that silk garments for AE are not cost-effective using currently accepted thresholds. At a willingness to pay of £30,000 per QALY, the probability of silk garments being cost-effective was 12.13%. This conclusion did not change in sensitivity analysis testing an alternative approach to costing the silk garments. Although the cost of silk garments was reduced with the alternative approach, at £53,989 per QALY, the estimated incremental cost per QALY was still over the accepted NICE threshold value (see S8 Table).

## Discussion

### Main findings

This trial found little evidence of clinical or economic benefit of using silk garments in addition to standard care, compared with standard care alone, in children with moderate to severe eczema. There were no differences between the treatment groups for any of the outcomes that were assessed by research nurses, who were unaware of participants’ treatment allocation, and the percentage of days on which topical corticosteroids or calcineurin inhibitors were used did not differ between the groups. The 95% confidence intervals around the primary efficacy estimates were narrow, suggesting that a clinically important treatment effect is unlikely to have been missed, and sensitivity analyses (imputing missing values, adjusting for baseline imbalances, and exploring the impact of adherence in wearing the garments) supported the primary analysis.

Subgroup analysis based on *FLG* genotype showed no evidence of differential treatment response in children with an inherited impairment in skin barrier function, and a post hoc analysis exploring the impact of baseline eczema severity on the primary outcome showed no effect, suggesting that children with more severe disease were no more likely to benefit from silk clothing than those with milder disease.

The trial garments are marketed as possessing antimicrobial properties, but this study found no evidence to suggest a reduction in the number of skin infections in those using the clothing compared to those randomised to standard care alone.

Of the seven unblinded secondary outcomes, two (POEM and PGA) showed small differences in favour of the silk garments, most noticeably in the first 3 mo of the trial. Whilst these small differences could have been genuine, they are most likely due to an expectation bias that declined with time. Our nested qualitative study (to be reported separately) highlighted the hopes that both children and parents placed on the silk clothing [11]. A previous eczema trial reported differences between blinded and unblinded outcomes when expectation regarding the benefits of the trial intervention was high [36].

### Relevance to other studies

To our knowledge, there have been no further RCTs on the effectiveness of silk garments for eczema since the CLOTHES Trial began (search updated 14 March 2016), and meta-analysis of the available silk clothing trials is not possible due to heterogeneity of designs. Additional brands of silk garments have since become available for use in eczema (e.g., Skinnies), but these have not been formally evaluated in RCTs. At the time of commissioning this research (2011), £840,272 was spent on prescriptions for silk garments per annum in the UK (for all indications). By 2014, this amount had risen to £2,082,810 per annum [37–40].

### Strengths and limitations

The CLOTHES Trial was an adequately powered RCT, with high follow-up rates and good adherence. The pragmatic study design meant that use of silk garments was evaluated as they might be used in normal practice, with mixed patterns of adherence. The trial placed special emphasis on objective outcome measures in order to minimise response bias.

It is possible that our emphasis on objective eczema severity outcomes meant that some important potential benefits were not captured in the primary analysis. Other factors, such as improvements in quality of life or a reduction in symptoms (especially itch and sleep loss, as measured by POEM), may be important drivers in determining whether or not patients feel that the garments are helpful. Nevertheless, we found no evidence of improved quality of life amongst trial participants using a range of validated scales.

Eczema severity scores improved for both groups during the trial, probably due to a combination of regression to the mean and regular monitoring of the eczema resulting in enhanced adherence to standard care. It is possible that treatment effects were masked by these general trial effects.

### Generalisability

The study has strong external validity as it was pragmatic in design to reflect normal clinical practice, and participants were recruited from five UK medical centres covering a range of urban and rural settings. We recruited children with a range of eczema severities, but the majority had moderate to severe disease; 32% had at least one mutation in the *FLG* gene, a proportion typical of eczema patents with moderate or severe disease [36]. Overall, 49% had self-reported food allergy, which is high for children with moderate to severe disease, and 15% reported a history of anaphylaxis. However, these data were collected by self-report and so may include food intolerance as well as food allergy.

We are unable to comment on the effectiveness of the silk garments if used continuously day and night, although sensitivity analysis found no evidence of improved outcomes in those who adhered more fully in wearing the garments. It is also possible that the beneficial effects of silk garments are best realised during a period of eczema flare, and daily use of the garments in the CLOTHES trial could have led to more rapid deterioration of the clothing than might have been seen if the garments were worn occasionally when the eczema was at its worst.

### Conclusion

This is the first large, independent trial to have evaluated silk garments for the management of eczema. The nested economic evaluation suggests that use of these garments is unlikely to be cost-effective for health providers, even if the small observed benefits were genuine. These trial results provide health commissioners with a better evidence base on which to make informed decisions about silk garments for eczema. Whether or not parents feel that the small benefits identified in some of the secondary outcomes are sufficient to justify purchasing these garments is something for individuals to consider on a case-by-case basis.

## Supporting information

S1 Alternative Language Abstract French translation of the abstract by Sébastien Barbarot. (DOCX)

S2 Alternative Language Abstract Chinese translation of the abstract by Lu Ban. (DOC)

S3 Alternative Language Abstract Spanish translation of the abstract by Ignacio Garcia Doval. (DOCX)

S4 Alternative Language Abstract Japanese translation of the abstract by Masaki Futamura. (DOCX)

S5 Alternative Language Abstract German translation of the abstract by Christien Apfelbacher and Uwe Matterne. (DOCX)

S1 Table Unit costs in 2014–2015 UK pounds sterling. (DOCX)

S2 Table Adherence according to age and baseline severity of eczema, plus sensitivity analysis for adherence. (DOCX)

S3 Table Sensitivity analyses adjusting for variables with baseline imbalance and imputation for missing primary outcome scores. (DOCX)

S4 Table Subgroup analysis of primary outcome by *FLG* status. (DOCX)

S5 Table Post hoc subgroup analysis for primary Eczema Area and Severity Index outcome according to baseline eczema severity. (DOCX)

S6 Table Mean (standard deviation) resource use and mean difference in resource use per participant (95% confidence interval). (DOCX)

S7 Table Mean (standard deviation) cost and cost difference (95% confidence interval) per participant over the 6 mo (in 2014–2015 UK pounds sterling). (DOCX)

S8 Table Incremental cost-effectiveness analyses results for base case and sensitivity analysis testing an alternative approach to costing silk garments. (DOCX)

S1 Protocol CLOTHES protocol final v3.0_11 (February 2014). (PDF)

S1 Checklist CONSORT checklist. (DOC)

## Abbreviations

ADQoL: Atopic Dermatitis Quality of Life
CACE: complier average causal effect
CHU9D: Child Health Utility 9 Dimensions
DFI: Dermatitis Family Impact
EASI: Eczema Area and Severity Index
HOME: Harmonising Outcomes Measures for Eczema
IGA: Investigator Global Assessment
NHS: National Health Service
NICE: National Institute for Health and Care Excellence
PGA: participant global assessment
POEM: Patient Oriented Eczema Measure
PPI: public and patient involvement
QALY: quality-adjusted life year
RCT: randomised controlled trial
SAE: serious adverse event
SD: standard deviation
TIS: Three Item Severity
