# Supplementary material for: Silk garments plus standard care compared with standard care for treating eczema in children: A randomised, controlled, observer-blind, pragmatic trial (CLOTHES Trial)
Source: PLoS Med. 2017 Apr 11;14(4):e1002280. doi: 10.1371/journal.pmed.1002280 (PMC5388469; doi:10.1371/journal.pmed.1002280)
Supplement: S1 Alternative Language Abstract — (DOCX) [file pmed.1002280.s001.docx]

**Efficacité des vêtements de soie associés à une prise en charge standard comparé à une prise en charge standard seule dans le traitement de la dermatite atopique: essai randomisé contrôlé pragmatique en insu de l’évaluateur.**

**Background**

La place des vêtements thérapeutiques dans la prise en charge de la dermatite atopique (eczéma atopique) est mal connue. Cette étude a évalué l’efficacité et le rapport coût/bénéfice du port de vêtements de soie dans la prise en charge de la dermatite atopique modérée à sévère de l’enfant.

**Methods and findings**

Il s’agissait d’un essai randomisé en groupe parallèle en insu de l’évaluateur. Les enfants, âgés de 1 à 15 ans, étaient recrutés auprès de médecins généralistes ou de spécialistes dans 5 centres au Royaume-Uni.

Les participants étaient répartis par une méthode de randomisation en ligne (1: 1) dans le bras du traitement standard seul ou dans le bras du traitement standard associé au port de vêtements en soie. Les participants étaient stratifiés par âge et par centre de recrutement. Les vêtements en soie étaient portés pendant 6 mois.

Le critère de jugement principal était la sévérité de la dermatite atopique, évalué par des infirmières en insu du bras d’allocation, à l’inclusion et à 2, 4 et 6 mois à l'aide du score de gravité EASI (résultats transformés en log pour l’analyse en intention de traiter). Le critère de jugement secondaire évaluant l’innocuité de l’intervention était le nombre d’infections cutanées observé pendant l’étude.

Trois cents enfants ont été randomisés (du 26 novembre 2013 au 5 mai 2015): 42% étaient des filles, 79% étaient de type caucasien, l’âge moyen était de 5 ans. L'analyse primaire comprenait 282/300 enfants (94%) (n = 141 dans chaque groupe). Les vêtements de soie étaient portés plus souvent la nuit [médiane 81% (25e au 75e percentile : 57% à 96%)] que le jour [médiane 34% (25e à 75e centile : 10% à 76%)].

Les moyennes géométriques des scores EASI à l’inclusion et à 2, 4 et 6 mois étaient respectivement de 9,2 ; 6,4 ; 5,8  et 5,4 pour le groupe des vêtements de soie et de 8,4; 6,6; 6,0 et 5,4 pour le groupe traitement standard seul. La moyenne globale du score EASI au cours de l’étude n’était pas statistiquement différente dans les 2 groupes (ratio ajusté des moyennes géométriques: 0,95, IC 95% 0,85 à 1,07 [la moyenne était ajustée sur le score EASI à l’inclusion, l'âge et le centre]). Cet intervalle de confiance équivaut à une différence de -1,5 à 0,5 par rapport au score EASI à l’inclusion ce qui n'est pas cliniquement pertinent. Les infections cutanées étaient observées chez 36/142 (25%) participants du groupe des vêtements de soie et chez 39/141 (28%) participants du groupe traitement standard. Bien que mineur, l’effet thérapeutique observé était réel mais le coût supplémentaire par QALY (année-personne sans invalidité) était de £56,811 selon l'analyse du NHS. Ces résultats suggèrent que le rapport coût/bénéfice des vêtements de soie n’est probablement pas favorable selon les critères médico-économiques usuels. Principale limite de l’étude: Même si le choix d'un critère de jugement principal objectif est susceptible d’avoir limité les biais de mesure, il peut avoir induit une sous-estimation de l’effet thérapeutique.

**Conclusions**

L’utilisation de vêtements de soie n’apporte probablement pas de bénéfice supplémentaire au traitement standard des enfants atteints de dermatite atopique modérée à sévère.

Kindly translated by Dr Sébastien Barbarot
