# Supplementary material for: Silk garments plus standard care compared with standard care for treating eczema in children: A randomised, controlled, observer-blind, pragmatic trial (CLOTHES Trial)
Source: PLoS Med. 2017 Apr 11;14(4):e1002280. doi: 10.1371/journal.pmed.1002280 (PMC5388469; doi:10.1371/journal.pmed.1002280)
Supplement: S2 Alternative Language Abstract — (DOC) [file pmed.1002280.s002.doc]

**丝绸服装加上标准护理与单标准护理在治疗儿童湿疹效果上的比较：一个随机对照，观察者盲法，实用试验（CLOTHES试验）**

**摘要**

**研究背景**

我们对服装在治疗湿疹（异位性皮炎，特应性湿疹）的作用了解甚少。本试验评估了丝绸服装（除了标准护理之外）对中度至重度湿疹患儿治疗的有效性和成本效益。

**研究方法和研究结果**

这是一个平行组随机对照，观察者盲法试验。 我们从英国五个包括二级医疗和社区医疗的研究中心招募1至15岁的患有中度至重度湿疹的患儿。

参与者使用在线随机（1：1）的方法被分配到单标准护理组或标准护理加丝绸服装组; 按年龄和招募中心分层。标准护理加丝绸服装组的湿疹患儿被要求穿丝绸服装6个月。

对治疗分配不知情的护士分别在基线和第2，4和6个月使用湿疹面积和严重程度指数（EASI）对本试验的主要结果（湿疹严重性）进行评价。该指数被对数转换以用于研究结果的分析（意向性治疗分析）。安全性结果：皮肤感染的数量。

三百名中度至重度湿疹患儿被随机分组​​（2013年11月26日至2015年5月5日）：42％是女孩，79％是白人，平均年龄是5岁。主要分析结果包括了282/300（94％）患儿（每组141例）。在这些患儿中，晚上穿丝绸服装的比白天穿的更多（在晚上：中间值81％（25％至75％区间值57％至96％）；在白天：34％（25％至75％区间值10％至76％）。在基线和第2，4和6个月时，标准护理加丝绸服装组的几何平均EASI分数为9.2，6.4，5.8和5.4，单标准护理组的几何平均EASI分数为8.4，6.6，6.0和5.4。经过在数据分析中调整不同基线EASI评分，患儿年龄和试验中心可能存在的潜在干扰作用，我们没有发现证据表明标准护理加丝绸服装组与单标准护理组在所有随访中得到的平均EASI评分有显著统计学差异（几何平均值的调整比例：0.95，95％置信区间 0.85至1.07）。该置信区间相等于在原始EASI标度单位中的-1.5至0.5的差异。该差异没有重要的临床意义。标准护理加丝绸服装组与单标准护理组皮肤感染的发生率分别为36/142（25％）和39/141（28％）。在从NHS角度的基础案例分析中，每个QALY的增量成本为56811英镑。这表明即使观察到的微小治疗效果是真实的丝绸服装在当前NHS可接受的阈值内不可能具有成本效益。试验的主要局限：虽然最大降低了检测偏倚，但对主要结果使用客观的评价标准可能会低估了治疗效果。

**研究结论**

与标准护理相比，丝绸服装很可能没有对患有中度至重度湿疹的患儿的湿疹治疗提供额外的好处。

Translation kindly provided by Dr Lu Ban
