# Supplementary material for: Silk garments plus standard care compared with standard care for treating eczema in children: A randomised, controlled, observer-blind, pragmatic trial (CLOTHES Trial)
Source: PLoS Med. 2017 Apr 11;14(4):e1002280. doi: 10.1371/journal.pmed.1002280 (PMC5388469; doi:10.1371/journal.pmed.1002280)
Supplement: S3 Alternative Language Abstract — (DOCX) [file pmed.1002280.s003.docx]

**Ropa de seda y tratamiento convencional, comparado con tratamiento convencional del eccema atópico infantil: ensayo clínico pragmático, aleatorizado, controlado, con enmascaramiento del observador (Ensayo CLOTHES).**

## Antecedentes

Sabemos poco del papel de la ropa en el tratamiento del eccema atópico (dermatitis atópica). Este ensayo clínico valoró la efectividad y eficiencia de la ropa de seda, añadida al tratamiento convencional, para el eccema atópico infantil moderado o grave.

## Métodos y resultados

Ensayo clínico aleatorizado, con dos grupos paralelos y cegamiento del observador. Se reclutaron niños entre 1 y 15 años de edad, con eccema atópico moderado o grave, en atención secundaria, primaria o desde la comunidad en cinco centros de Reino Unido.

Los participantes se aleatorizaron (1:1) mediante un sistema on-line para recibir tratamiento convencional o tratamiento convencional y ropa de seda, con estratificación por edad y centro. Las prendas de seda se utilizaron durante 6 meses.

El resultado principal (gravedad del eccema) lo valoraron al inicio y a los 2, 4 y 6 meses enfermeras desconocedoras del tratamiento recibido, empleando el *Eczema Area and Severity Index* (EASI). Para el análisis (por intención de tratar) se utilizó una trasformación logarítmica del EASI. Como resultado de seguridad se midió el número de infecciones cutáneas.

Se aleatorizaron trescientos niños (26 noviembre de 2013 a 5 de mayo de 2015): 42% niñas, 79% de raza blanca, y con una edad media de 5 años. El análisis principal incluyó 282/300 (94%) niños (n=141 en cada grupo). Las prendas se utilizaron más frecuentemente de noche (mediana: 81% de las noches (percentiles 25- 75: 57% a 96%) y 34% de los días (percentiles 25- 75: 10% a 76%)). Las medias geométricas del índice EASI al inicio y en los meses 2, 4, y 6 fueron: 9.2, 6.4, 5.8, 5.4 en los usuarios de prendas de seda y 8.4, 6.6, 6.0, 5.4 en los no usuarios. No encontramos diferencias entre los grupos para la media de EASI de todas las visitas de seguimiento, ajustadas por el EASI basal, edad y centro (cociente ajustado de las medias geométricas: 0.95, IC95% 0.85 a 1.07). Este intervalo de confianza equivale a una diferencia de -1.5 a 0.5 unidades originales de EASI, que es clínicamente irrelevante. Presentaron infecciones cutáneas 36/142 (25%) de los usuarios de ropa de seda y 39/141 (28%) de los no usuarios. Aún en el caso de que el pequeño efecto observado fuese real, el coste incremental por año de vida ajustado por calidad (QUALY, AVAC) sería de £56,811, desde la perspectiva del NHS, lo que indica que las prendas de seda no se considerarían eficientes con los umbrales actualmente aceptados. La limitación principal del estudio es que el uso de una medida objetiva del efecto, aunque minimiza el sesgo de detección, podría infra-estimar el efecto del tratamiento.

## Conclusiones

Es muy poco probable que añadir ropa de seda al tratamiento convencional del eccema atópico infantil moderado o grave suponga un beneficio.

## Registro del ensayo clínico

El ensayo clínico se registró en *Current Controlled Trials* antes del inicio del reclutamiento (ISRCTN77261365 11 Oct 2013)

Kindly translated by Dr Ignacio Garcia Doval
