# Supplementary material for: Silk garments plus standard care compared with standard care for treating eczema in children: A randomised, controlled, observer-blind, pragmatic trial (CLOTHES Trial)
Source: PLoS Med. 2017 Apr 11;14(4):e1002280. doi: 10.1371/journal.pmed.1002280 (PMC5388469; doi:10.1371/journal.pmed.1002280)
Supplement: S4 Alternative Language Abstract — (DOCX) [file pmed.1002280.s004.docx]

**小児アトピー性皮膚炎に対する標準治療へのシルク肌着着用の併用効果：無作為化評価者盲検実用的臨床試験（CLOTHES試験）**

## 背景

アトピー性皮膚炎の管理上、衣類が果たす役割はよく知られていない。本試験では中等症～重症の小児アトピー性皮膚炎管理における（標準治療に併用した）シルク肌着の効果と費用対効果を検討した。

## 方法と結果

本試験は、評価者盲検による無作為化並行群間比較試験で行った。中等症～重症のアトピー性皮膚炎の1～15歳の子供が、英国5施設で二次医療施設やコミュニティーから集められた。

参加者は年齢と登録施設で階層化され、標準治療または標準治療とシルク肌着着用のいずれかにオンライン無作為化で1:1に割り付けられた。シルク肌着は6ヶ月間着用した。

主要評価項目（湿疹重症度）は、介入前、2、4、6か月後に治療割付を盲検化された看護師がEczema Area and Severity Index （EASI）で評価し、その対数変換したものを解析（intention-to-treat解析）に用いた。安全性として皮膚感染の回数を評価した。

2013年11月26日～2015年5月5日に300人の子供が無作為化割付され、42%が女児、79%が白人、平均年齢5歳であった。一次解析は282人（94%）の小児（各群141人）で行った。肌着は日中よりも夜間によく着用され、中央値で夜間の81%（第1四分位～第3四分位：57%～96%）、日中の34%（同10%～76%）に着用された。介入前、2、4、6ヶ月後の平均EASIスコアは、シルク肌着群では9.2、6.4、5.8、5.4、標準治療群では8.4、6.6、6.0、5.4であった。介入前のEASI、年齢、登録施設で調整したフォロー期間全体の平均EASIに両群間の差は認められなかった。（幾何平均の調整比0.95、95%信頼区間 0.85～1.07）。この信頼区間は、臨床的に重要でないとされるEASIの値-1.5～0.5の差に相当する。皮膚感染は、シルク肌着群と標準治療群でそれぞれ36/142（25%）と39/141（28%）に認められた。わずかな治療効果が真実であったとしても、NHS（英国国民健康保険）の立場からみたベースケース分析では質調整生存年（QALY）あたりの増分費用は56,811ポンドとなり、現在の基準で判定するとシルク肌着は費用対効果がよくないと考えられる。本試験の主な限界として、検出バイアスは最小限に抑えたが客観的な主要評価項目を使用したため治療効果が過小評価された可能性はある。

## 結論

中等症～重症の小児アトピー性皮膚炎に対して、標準治療へシルク肌着着用を併用することによる効果はなさそうである。

## 試験登録

本試験は、募集開始前にCurrent Controlled Trialに事前登録された（ISRCTN77261365、2013年10月11日）。

Translation kindly provided by Dr Masaki Futamura MD, Ph.D.

独）国立病院機構　名古屋医療センター

小児科 / アレルギー科

〒460-0001　名古屋市中区三の丸4-1-1

------------------

Chief, Division of Pediatrics and Division of Allergy

Nagoya Medical Center

4-1-1 Sannomaru, Naka-ku, Nagoya 460-0001, JAPAN
