# Supplementary material for: Silk garments plus standard care compared with standard care for treating eczema in children: A randomised, controlled, observer-blind, pragmatic trial (CLOTHES Trial)
Source: PLoS Med. 2017 Apr 11;14(4):e1002280. doi: 10.1371/journal.pmed.1002280 (PMC5388469; doi:10.1371/journal.pmed.1002280)
Supplement: S5 Alternative Language Abstract — (DOCX) [file pmed.1002280.s005.docx]

**Seidenkleidung plus gewöhnliche Versorgung im Vergleich zu gewöhnlicher Versorgung in der Behandlung des atopischen Ekzems bei Kindern: eine randomisierte, kontrollierte, Untersucher-verblindete, pragmatische Studie (CLOTHES - Studie)**

## Hintergrund

Es ist nur wenig dazu bekannt, welche Rolle Kleidung im Management des atopischen Ekzems (Synonym: Neurodermitis) spielt. In dieser Studie wurde die Wirksamkeit und Kosteneffektivität von Kleidung aus Seide (zusätzlich zu gewöhnlicher Versorgung) im Management des mittelschweren bis schweren atopisches Ekzems bei Kindern untersucht.

## Methoden und Ergebnisse

Es handelte sich um eine randomisierte, kontrollierte, Untersucher-verblindete Parallelgruppenstudie. Kinder im Alter von 1-15 Jahren mit mittelschwerem bis schwerem atopischen Ekzem wurden aus der Bevölkerung sowie über Allgemein- und Fachärzte rekrutiert. Die Zuteilung der Teilnehmer zu gewöhnlicher Versorgung beziehungsweise gewöhnlicher Versorgung plus Seidenkleidung erfolgte mittels Online-Randomisierung (1:1), stratifiziert nach Alter und Rekrutierungszentrum. Die Seidenkleidung wurden sechs Monate lang getragen.

Das primäre Outcome (Ekzemschwere) wurde bei Baseline sowie nach 2, 4 und 6 Monaten mittels „Eczema Area and Severity Index“ (EASI) durch Pflegekräfte erfasst, die gegenüber der Zuweisung der Behandlung verblindet waren. Der EASI wurde für die Analyse log-transformiert (Intention-to-Treat Analyse). Sicherheits-Outcome: Anzahl der Hautinfektionen.

Es wurden dreihundert Kinder randomisiert (26.November 2013 bis 05.Mai 2015): 42% Mädchen, 79% weiß, mittleres Alter 5 Jahre. Die Hauptanalyse wurde für 282/300 (94%) Kinder (n= 141 in jeder Gruppe) gemacht. Die Kleidungsstücke wurden häufiger nachts als am Tag getragen (Median 81% der Nächte (25. – 75. Perzentil 57% - 96%) und 34% der Tage (25.-75.Perzentil 10% - 76%)). Der geometrische EASI – Mittelwert bei Baseline sowie nach 2, 4 und 6 Monaten betrug 9.2, 6.4, 5.8, 5.4 für die Seidenkleidung und 8.4, 6.6, 6.0, 5.4 für gewöhnliche Versorgung. Zwischen den Gruppen zeigten sich keine Unterschiede in den über die Nachbeobachtungszeitpunkte gemittelten EASI-Werten, die für Baseline-EASI, Alter und Zentrum adjustiert waren (Adjustiertes Verhältnis der geometrischen Mittelwerte: 0.95, 95 % Konfidenzintervall (KI) 0.85 – 1.07). Dieses Konfidenzintervall entspricht einem Unterschied von -1,5 bis 0,5 EASI-Werten, was klinisch nicht bedeutsam ist. Hautinfektionen traten bei 36/142 (25%) und 39/141 (28%) der Kinder mit Seidenkleidung bzw. gewöhnlicher Versorgung auf. Auch wenn der kleine beobachtete Effekt echt wäre, lägen die inkrementellen Kosten pro QALY in der Basisanalyse aus der Perspektive des NHS bei £56,811. Es erscheint also unwahrscheinlich, dass Seiden-Kleidung innerhalb akzeptierter Grenzwerte kosteneffektiv ist. Hauptlimitationen: während durch einen objektiven primären Endpunkt ein Detektions-Bias minimiert wurde, könnten Behandlungseffekte unterschätzt worden sein.

## Schlussfolgerung

Es ist unwahrscheinlich, dass Seidenkleidung bei Kindern mit mittelschwerem bis schwerem atopischen Ekzem einen über die gewöhnliche Versorgung hinausreichenden Zusatznutzen hat.

## Studienregistrierung

Die Studie war vor Beginn der Rekrutierung bei Current Controlled Trials registriert (ISRCTN77261365 11 Oct 2013).

Kindlt translated by Dr Christien Apfelbacher and Dr Uwe Matterne
