# Supplementary material for: Silk garments plus standard care compared with standard care for treating eczema in children: A randomised, controlled, observer-blind, pragmatic trial (CLOTHES Trial)
Source: PLoS Med. 2017 Apr 11;14(4):e1002280. doi: 10.1371/journal.pmed.1002280 (PMC5388469; doi:10.1371/journal.pmed.1002280)
Supplement: S1 Table — (DOCX) [file pmed.1002280.s006.docx]

**S1 Table: Unit costs in 2014/15 UK pounds sterling**

| **Resource Item** | **Unit cost** | **Source^ref no.^** |
| --- | --- | --- |
| **Intervention**  Silk therapeutic garments  Base case: Prescription Cost Analysis Approach (per set)  Sensitivity Analysis: Tariff Approach (per set)  Silk therapeutic garments (Sensitivity analysis – NHSBSA actual cost) | £66.02 to £155.49  £62.83 to £145.02 | PCA^30^  Tariff^34^ |
| **Primary health care**  GP (Per surgery consultation)  GP (Per telephone consultation)  GP (Per consultation out of Hours)  Practice nurse (per consultation)  Community eczema nurse (per consultation)  Community nurse (per consultation)  Pharmacist (Per contact)  Health visitor (Per contact)  Nutritionist (Per telephone contact)  Blood test (Per test)  Flu Vaccination (per vaccine and nurse time)  **Secondary health care**  A&E (per visit)  Outpatients first visit (dermatology, per consultation)  Consultant Eczema nurse (Per telephone consultation)  Eczema nurse (Per telephone contact)  Paediatric assessment  Inpatient stay for skin disorder without intervention (1 night)  Inpatient stay for skin disorder without intervention (3 nights)  Inpatient stay for skin disorder without intervention (4 nights  Homeopathic visit  Patch test (Per test)  **Medications**  Various | £37·00  £22·00  £68·91  £12·14  £38·00  £38·00  £14·67  £54·00  £82·66  £9·07  30·14  £93·00  £128·40  £161·03  £42·57  £299·51  £1185·48  £1756·10  £2267·63  £57·00  £91·81  £various | PSSRU^28^  PSSRU^28^  PSSRU^28^  PSSRU^28^  PSSRU^28^  PSSRU^28^  PSSRU^28^  PSSRU^28^  DH^29^  DH^29^  PCA^30^/PSSRU^28^  PSSRU^28^  DH^29^  DH^29^  DH^29^  DH^29^  DH^29^  DH^29^  DH^29^  *  DH^29^  PCA^30^ |

***Estimate based on the range of values provided on:** [**http://www.nhs.uk/Conditions/Homeopathy/Pages/Introduction.aspx**](http://www.nhs.uk/Conditions/Homeopathy/Pages/Introduction.aspx)

**Supplemental reference**

39. NHS Business Services Authority. Drug Tariff March 2015. Available from: <http://www.ppa.org.uk/edt/March_2015/mindex.htm> (Accessed 10th June 2016).
