# Supplementary material for: Silk garments plus standard care compared with standard care for treating eczema in children: A randomised, controlled, observer-blind, pragmatic trial (CLOTHES Trial)
Source: PLoS Med. 2017 Apr 11;14(4):e1002280. doi: 10.1371/journal.pmed.1002280 (PMC5388469; doi:10.1371/journal.pmed.1002280)
Supplement: S2 Table — (DOCX) [file pmed.1002280.s007.docx]

**S2 Table: Adherence according to age and baseline severity of eczema**

| *Spearman correlation coefficient* | Percentage of days that clothing was worn for at least some of the day  n=124 | Percentage of nights that clothing was worn for at least some of the night  n=124 |
| --- | --- | --- |
| Age | 0.003 | 0.20 |
| Baseline EASI score | -0.03 | 0.03 |
| Baseline POEM score | 0.08 | 0.13 |

Spearman correlation coefficients range between -1 (perfect negative correlation) and 1 (perfect postive correlation). Correlation coefficient of 0 indicates no correlation.

**Sensitivity analysis - adherence with trial garments making assumptions for missing data**

|  | Main analysis  (participants with 12 or more questionnaire completed )  (n = 124) | Sensitivity analysis 1^$^  (n = 149) | Sensitivity analysis 2^  (n = 149) |
| --- | --- | --- | --- |
|  |  |  |  |
| Proportion of nights that garments were worn for at least some of the night |  |  |  |
| Median [25^th^, 75^th^ centile] | 80.7 [56.8, 95.9] | 74.4 [52.1, 94.8] | 61.5 [32.9, 87] |
|  |  |  |  |
| Percentage of days that clothing were worn for at least some of the day |  |  |  |
| Median [25^th^, 75^th^ centile] | 34.1 [9.8, 75.9] | 28.6 [3.7, 74.3] | 19.3 [2.5, 63.4] |
|  |  |  |  |
| Adherence with trial garments |  |  |  |
| Adherent^a^ | **102 (82%)** | **117 (79%)** | **87 (58%)** |
| Worn for at least 50% of days & 50% of nights | 50 (40%) | 54 (36%) | 45 (30%) |
| Worn for at least 50% of days only | - | 1 (1%) | - |
| Worn for at least 50% of nights only | 52 (42%) | 62 (42%) | 42 (28%) |
|  |  |  |  |
| Not adherent | **22 (18%)** | **32 (21%)** | **62 (42%)** |
|  |  |  |  |

Adherence with trial garments summarised from week 2 onwards.

a - Participant were defined as adherent with trial garments if they were worn for at least 50% of the days or at least 50% of the nights.

Both sensitivity analyses include all participants in the interventon group regardless of the number of questionnaires completed and assumes that participants never wore the garments if they did not complete any questionnaires about how often they wore the garments (5 participants).

^$^Sensitivity analysis 1 - assuming garments worn for the same proportion of time when the questionnaire wasn’t completed as when the questionnaires were returned.

^Sensitivity analysis 2 – assuming garments not worn when the questionnaire wasn’t completed
