# Supplementary material for: Silk garments plus standard care compared with standard care for treating eczema in children: A randomised, controlled, observer-blind, pragmatic trial (CLOTHES Trial)
Source: PLoS Med. 2017 Apr 11;14(4):e1002280. doi: 10.1371/journal.pmed.1002280 (PMC5388469; doi:10.1371/journal.pmed.1002280)
Supplement: S3 Table — (DOCX) [file pmed.1002280.s008.docx]

**S3 Table: Sensitivity analysis for primary outcome**

|  | Ratio of geometric means (95% CI) |
| --- | --- |
|  |  |
| **Sensitivity analysis 1:** |  |
| Additionally adjusting for baseline imbalance in gender, history of asthma and history of food allergy^*^ | n = 282  0·95 (0·85, 1·07) |
|  |  |
| **Sensitivity analysis 2:** |  |
| Using multiple imputation for missing outcomes^$^ | n = 300 |
| Assuming missing EASI scores missing at random (MAR) | 0·93 (0·83, 1·05) |
|  |  |
| *Assuming missing EASI scores are missing not at random* |  |
| *Favouring intervention group* |  |
| Assuming EASI scores are 3 points higher (worse) than under MAR in the standard care group & MAR in intervention group | 0·89 (0·80, 1·01) |
|  |  |
| *Favouring standard care group* |  |
| Assuming EASI scores are 3 points higher than under MAR in the intervention group & MAR in standard care group | 0·97 (0·86, 1·09) |
|  |  |
| **Sensitivity analysis 3:** |  |
| Comparison of EASI scores at 6 month follow-up† | n = 243 |
| According to allocated group, ignoring adherence^1^ | 1.026 (0.87, 1.21) |
|  |  |
| CACE using binary definition of adherence that garments worn for at least 50% of days or 50% of the nights | 1.031 (0.85, 1.25) |
|  |  |
| CACE for each additional 10% of time garments worn | 1.004 (0.977, 1.032) |
|  |  |

*** Gender, history of asthma and history of food allergy were chosen due to slight imbalance at baseline.**

**$ - Multiple imputation using chained equations. The imputation model included age, site, gender, POEM scores at the clinic visits, whether there had been a treatment escalation during the study and mean POEM score from the weekly questionnaires (as well as number of questionnaires included). 20 datasets were imputed and estimates were combined using Rubin rules.**

**†- For participants who reported adherence in at least 12 or more questionnaires (n = 122 participants in the standard care group and n = 121 in the intervention group)**

**1- Analysed using linear regression with log transformed EASI score at 6 months as the outcome variable and adjusted for randomisation stratification variables and baseline EASI score.**
