# Supplementary material for: Silk garments plus standard care compared with standard care for treating eczema in children: A randomised, controlled, observer-blind, pragmatic trial (CLOTHES Trial)
Source: PLoS Med. 2017 Apr 11;14(4):e1002280. doi: 10.1371/journal.pmed.1002280 (PMC5388469; doi:10.1371/journal.pmed.1002280)
Supplement: S4 Table — (DOCX) [file pmed.1002280.s009.docx]

**S4 Table: Subgroup analysis for primary EASI outcome of eczema severity according to filaggrin genotype (none, one, or two *FLG* null mutations) for participants of white European ethnicity**

| Subgroup  Allocated group | Baseline | 2 months | 4 months | 6 months | Subgroup specific ratio of geometric means (95% CI) | Interaction effect^$^  (95% CI) | p-value for interaction effect |
| --- | --- | --- | --- | --- | --- | --- | --- |
|  |  |  |  |  |  |  |  |
| ***FLG* wild type; no mutations** | |  |  |  |  |  |  |
| **Standard care**  Median [25th, 75th centile]  Geometric mean | (n = 72)  6·2 [3·9, 10·7]  7·7 | (n = 67)  4·5 [2·4, 9·0]  6·1 | (n = 65)  3·2 [2·1, 9·9]  5·5 | (n = 69)  3·3 [1·8, 6·8]  4·8 | 1·04 (0·89, 1·21) |  |  |
| **Intervention**  Median [25th, 75th centile]  Geometric mean | (n = 71)  5·4 [3·3, 13·8]  8·1 | (n = 67)  4·3 [2·1, 10·3]  6·4 | (n = 67)  3·8 [2·2, 8·4]  5·7 | (n = 67)  4·0 [2·3, 9·9]  6·1 |  |  |  |
|  |  |  |  |  |  | 0·84 (0·61, 1·15) |  |
| **One *FLG* null mutation** | |  |  |  |  |  |  |
| **Standard care**  Median [25th, 75th centile]  Geometric mean | (n = 31)  8·0 [3·8, 12·0]  8·5 | (n = 28)  5·3 [2·9, 11·4]  7·1 | (n = 27)  4·6 [2·7, 8·6]  6·5 | (n = 29)  4·4 [1·6, 10·7]  5·4 | 0·87 (0·67, 1·14) |  |  |
| **Intervention**  Median [25th, 75th centile]  Geometric mean | (n = 20)  8·7 [5·0, 15·7]  10·1 | (n = 19)  6·1 [3·0, 8·4]  6·9 | (n = 19)  4·4 [2·2, 9·5]  5·5 | (n = 19)  4·0 [1·9, 8·0]  5·2 |  |  |  |
|  |  |  |  |  |  |  | 0·47 |
| **Two *FLG* null mutations** | |  |  |  |  |  |  |
| **Standard care**  Median [25th, 75th centile]  Geometric mean | (n = 12)  17·9 [7·7, 23·4]  13·7 | (n = 11)  10·7 [3·8, 23·6]  10·3 | (n = 12)  10·8 [3·6, 16·1]  9·3 | (n = 12)  9·9 [4·1, 14·3]  9·9 | 0·89 (0·60, 1·30) | 0·85 (0·56, 1·29) |  |
| **Intervention**  Median [25th, 75th centile]  Geometric mean | (n = 11)  12·4 [8·6, 16·6]  13·0 | (n = 11)  6·6 [5·4, 16·8]  8·9 | (n = 9)  9·3 [5·3, 23·4]  10·2 | (n = 9)  7·4 [2·6, 16·5]  7·8 |  |  |  |
|  |  |  |  |  |  |  |  |

**^$^Ratio of geometric means for intervention versus standard care in individuals with one or two *FLG* null mutations compared to ratio of geometric means for intervention versus usual care for *FLG* wild type genotype. 209 participants were included in the analysis model (n = 110 standard care, 99 intervention).**

**We also compared the *FLG* wild type genotype with the combined group having one or two *FLG* null mutations in a further exploratory analysis: the interaction effect for any mutation compared to no mutation was 0.85 (95% CI 0.65 to 1.11).**
