# Supplementary material for: Silk garments plus standard care compared with standard care for treating eczema in children: A randomised, controlled, observer-blind, pragmatic trial (CLOTHES Trial)
Source: PLoS Med. 2017 Apr 11;14(4):e1002280. doi: 10.1371/journal.pmed.1002280 (PMC5388469; doi:10.1371/journal.pmed.1002280)
Supplement: S5 Table — (DOCX) [file pmed.1002280.s010.docx]

**S5 Table: Post-hoc subgroup analysis for primary EASI outcome of eczema severity according to baseline eczema severity**

| **Subgroup**  **Allocated group** | Baseline | 2 month | 4 months | 6 months | Subgroup specific ratio of geometric means (95% CI) |
| --- | --- | --- | --- | --- | --- |
|  |  |  |  |  |  |
| **Almost clear/mild baseline EASI scores (1 to 7)** | |  |  |  |  |
| **Standard care**  n  Mean [sd]  Median [25th, 75th centile]  Geometric mean | 73  4.1 [1.7]  4 [2.6, 5.5]  4.8 | 66  4 [3.7]  2.9 [1.8, 4.8]  4.2 | 65  3.3 [2.5]  2.7 [1.5, 4]  3.7 | 68  3.4 [3.3]  2.3 [1.4, 4.3]  3.5 | 0.95 (0.81, 1.12) |
| **Intervention**  n  Mean [sd]  Median [25^th^, 75^th^ centile]  Geometric mean | 75  4.2 [1.6]  4.1 [2.8, 5.5]  4.9 | 72  3.6 [3.7]  2.8 [1.6, 4.3]  3.8 | 72  3.5 [3.1]  2.7 [1.6, 4.3]  3.7 | 70  3.7 [4.6]  2.5 [1.2, 4.2]  3.7 |  |
|  |  |  |  |  |  |
| **Moderate/severe baseline EASI scores (7.1 to 50)** | |  |  |  |  |
| **Standard care**  n  Mean [sd]  Median [25th, 75th centile]  Geometric mean | 78  14.7 [7.7]  12 [8.8, 18.8]  14.2 | 71  11.4 [7.9]  9.2 [5.2, 15.8]  10.0 | 68  12 [10.3]  9.4 [4.7, 16.4]  9.5 | 71  9.4 [7.3]  7.7 [4.1, 12.3]  8.2 | 0.95 (0.81, 1.12) |
| **Intervention**  n  Mean [sd]  Median [25^th^, 75^th^ centile]  Geometric mean | 74  18.8 [10.8]  15.4 [10, 23.1]  17.3 | 67  14.5 [12.6]  9.6 [5.6, 19.6]  11.3 | 63  12.6 [12.9]  8 [4, 17.3]  9.5 | 63  11.4 [12.6]  6.9 [3.9, 13.8]  8.3 |  |
|  |  |  |  |  |  |

Ratio of geometric means and the interaction effect adjusted for baseline EASI score and the stratification variables age and site as covariates

282 participants were included in the analysis model (n = 141 each group).

Coefficient from multilevel model from interaction term between baseline EASI (log transformed and continuous) and treatment group:

0.0827 95% CI -0.0801 to 0.2454, back transformed 1.086 95% CI 0.923 to 1.278, p-value 0.32

EASI eczema severity categories are based on those in Leshem, Y.A., Hajar, T., Hanifin, J.M. and Simpson, E.L. (2015), What the Eczema Area and Severity Index score tells us about the severity of atopic dermatitis: an interpretability study. British Journal of Dermatology, 172: 1353–1357. doi: 10.1111/bjd.13
