# Supplementary material for: Silk garments plus standard care compared with standard care for treating eczema in children: A randomised, controlled, observer-blind, pragmatic trial (CLOTHES Trial)
Source: PLoS Med. 2017 Apr 11;14(4):e1002280. doi: 10.1371/journal.pmed.1002280 (PMC5388469; doi:10.1371/journal.pmed.1002280)
Supplement: S6 Table — (DOCX) [file pmed.1002280.s011.docx]

**S6 Table: Mean (Standard Deviation) Resource Use and Mean Difference in Resource Use per Participant (95% Confidence Interval)**

| **Resource use item** | **Intervention (n=134)**  **Number (SD)** | **Standard care (n=139)**  **Number (SD)** | **Mean difference (95% CI)** |
| --- | --- | --- | --- |
| **Intervention** | | | |
| Silk therapeutic garments (number of sets provided over 6 months) | 4·15 (1·55) | 0·00 (0·00) | 4·15 (3·88, 4·41) |
| **Primary health care** | | | |
| GP (Per surgery consultation) | 0·78 (1·08) | 1·06 (1·74) | -0·29 (-0·64, 0·06) |
| GP (Per telephone consultation) | 0·01 (0·09) | 0·01 (0·12) | -0·007  (-0·03, 0·02) |
| GP (Per consultation out of Hours) | 0·00 (0·00) | 0·01 (0·06) | -0·01 (-0·02, 0·01) |
| Practice nurse (per consultation) | 0·13 (0·40) | 0·07 (0·35) | 0·06 (-0·03, 0·15) |
| Community eczema nurse (per consultation) | 0·03 (0·27) | 0·01 (0·08) | 0·02 (-0·02, 0·7) |
| Community nurse (per consultation at home) | 0·01 (0·09) | 0·00 (0·00) | 0·01 (-0·01, 0·02) |
| Pharmacist (Per contact) | 0·00 (0·00) | 0·01 (0·08) | -0·01 (-0·02, 0·01) |
| Health visitor (Per contact) | 0·00 (0·00) | 0·01 (0·09) | -0·01 (-0·3, 0·01) |
| Nutritionist (Per telephone contact) | 0·00 (0·00) | 0·01 (0·08) | -0·01 (-0·02, 0·01) |
| Homeopathic (per visit) | 0·01 (0·17) | 0·07 (0·55) | -0·06  (-0·15, 0·04) |
| Blood test (Per test) | 0·01 (0·09) | 0·01 (0·17) | -0·007  (-0·04, 0·03) |
| Flu vaccination | 0·00 (0·00) | 0·01 (0·06) | -0·01 (-0·02, 0·01) |
| **Total number of primary care visits** | **0·98 (1·17)** | **1·28 (1·87)** | **-0·30**  **(-0·68, 0·07)** |
| **Secondary health care** | | | |
| A&E (per visit) | 0·01 (0·17) | 0·01 (0·12) | 0·0005  (-0·03, 0·04) |
| Outpatients first visit (dermatology, per consultation) | 1·04 (1·69) | 0·83 (1·50) | 0·21 (-0·17, 0·59) |
| Dermatology consultation (per phone call or email contact) | 0·01 (0·17) | 0·01 (0·11) | 0·001 (-0·03, 0·04) |
| Consultant Eczema nurse (Per telephone consultation) | 0·00 (0·00) | 0·01 (0·08) | -0·01 (-0·02, 0·01) |
| Eczema nurse (Per telephone contact) | 0·00 (0·00) | 0·01 (0·08) | -0·01 (-0·02, 0·01) |
| Paediatric assessment unit | 0·00 (0·00) | 0·02 (0·19) | -0·02 (-0·5, 0·01) |
| Children’s ward (Number of visits) | 0·04 (0·36) | 0·03 (0·34) | 0·01 (-0·07, 0·09) |
| Inpatient stay for skin disorder without intervention | 0·04 (0·27) | 0·02 (0·19) | 0·02 (-0·03, 0·08) |
| Patch test (Per test) | 0·00 (0·00) | 0·02 (0·25) | -0·2 (-0·06, 0·02) |
| **Total number of secondary care visits** | **1·16 (2·12)** | **0·98 (1·65)** | **0·18 (-0·27, 0·63)** |
| **Total number of health care visits** | **2·13 (2·79)** | **2·26 (2·55)** | **-0·12**  **(-0·76, 0·51)** |
| **Medications** | | | |
| Prescription items (number, including TCS) | 12·56 (17·97) | 12·60 (13·90) | -0·04 (-3·86, 3·78) |
| Topical corticosteroid (TCS) (g) | 139.03 (212.49) | 169.03 (295.14) | -30.00 (-91.47, 31.47) |
