# Supplementary material for: Silk garments plus standard care compared with standard care for treating eczema in children: A randomised, controlled, observer-blind, pragmatic trial (CLOTHES Trial)
Source: PLoS Med. 2017 Apr 11;14(4):e1002280. doi: 10.1371/journal.pmed.1002280 (PMC5388469; doi:10.1371/journal.pmed.1002280)
Supplement: S7 Table — (DOCX) [file pmed.1002280.s012.docx]

**S7 Table: Mean (Standard Deviation) Cost and Cost Difference (95% Confidence Interval) Per Participant over the 6 months (in 2014/15 UK pounds sterling)(Base case)**

| **Resource use item** | **Intervention (n=134):**  **mean (SD) £’s** | **Standard Care (n=139):**  **mean (SD) £’s** | **Mean difference**  **(95% CI) £’s** |
| --- | --- | --- | --- |
| **Intervention resource use – Silk therapeutic garments (including replacements)** | | | |
| Base case – Prescription Cost Analysis | 318.52 (136.60) | 0·00  (0·00) | 318.52  (295.71, 341.33) |
| Sensitivity analysis – Tariff Approach | 300.72 (119.52) | 0·00  (0·00) | 300.72  (280.76, 320.67) |
| **Primary health care** | | | |
| GP (surgery consultation) | 28·72 (39·98) | 39·40 (64·43) | -10·68  (-23·51, 2·15) |
| GP (telephone consultation) | 0·16 (1·90) | 0·32 (2·63) | -0·15 (-0·70, 0·40) |
| GP (consultation out of Hours) | 0·00 (0·00) | 0·50 (5·84) | -0·50 (-1·49, 0·50) |
| Practice nurse | 1·63 (4·89) | 0·87 (4·30) | 0·76 (-0·34, 1·85) |
| Community eczema nurse | 4·81 (43·89) | 1·16 (13·66) | 3·65 (-4·04, 11·34) |
| Community nurse | 0·28 (3·28) | 0·00 (0·00) | 0·28 (-0·26, 0·83) |
| Pharmacist | 0·00 (0·00) | 0·11 (1·24) | -0·11 (-0·32, 0·11) |
| Health visitor | 0·00 (0·00) | 0·78 (6·45) | -0·78 (-1·87, 0·32) |
| Nutritionist (telephone contact) | 0·00 (0·00) | 0·59 (7·01) | -0·59 (-1·78, 0·60) |
| Homeopathic visit | 0·85 (9·85) | 4·10 (31·18) | -3·25 (-8·80, 2·30) |
| Blood test | 0·07 (0·78) | 0·13 (1·54) | -0·06 (-0·36, 0·23) |
| Flu vaccination | 0·00 (0·00) | 0·22 (2·56) | -0·22 (-0·65, 0·22) |
| **Total Primary health care costs** | **36·52 (57·74)** | **47·01 (73·71)** | **-10·49**  **(-26·30, 5·33)** |
| **Secondary health care** | | | |
| A&E | 1·39 (16·07) | 1·34 (11·12) | 0·5 (-3·23, 3·33) |
| Outpatients first visit (dermatology consultation) | 134·15  (217·53) | 107·15 (192·82) | 27·00  (-21·94, 75·93) |
| Dermatologist consultant  (phone/email consultation) | 0·96 (11·09) | 0·92 (7·67) | 0·03 (-2·23, 2·30) |
| Consultant Eczema nurse (telephone consultation) | 0·00 (0·00) | 0·21 (2·46) | -0·21 (-0·63, 0·21) |
| Eczema nurse (telephone contact) | 0·00 (0·00) | 1·16 (13·66) | -1·16 (-3·48, 1·16) |
| Paediatric assessment unit | 0·00 (0·00) | 6·46 (56·64) | -6·46  (-16·10, 3·17) |
| Children’s ward | 11·18  (106·49) | 8·62 (101·62) | 2·56  (-22·24, 27·35) |
| Inpatient stay for skin disorder without intervention | 65·42 (401·7) | 24·84 (216·39) | 40·57  (-35·94, 117·08) |
| Patch test | 0·00 (0·00) | 1·98 (23·36) | -1·98 (-5·96, 1·99) |
| **Total secondary health care costs** | **213·09 (604·47)** | **153·00 (327·13)** | **60·09**  **(-55·16, 175·34)** |
| **Total prescription costs** | **119.82 (244.67)** | **120.86 (243.81)** | **-1.04 (-59.25, 57.18)** |
| **Mean total health care costs without silk garments** | **369.43 (805.88)** | **320.86 (446.13)** | **48.57 (-105.92, 203.05)** |
| **Mean total health care costs with silk garments (Base case - PCA)** | **687.96 (809.27)** | **320.86 (446.13)** | **367.09 (212.12, 522.07)** |
| **Mean total health care costs with silk garments (Sensitivity analysis – Tariff Approach)** | **670.15 (809.66)** | **320.86 (446.13)** | **349.28 (194.25, 504.32)** |
