# Supplementary material for: Silk garments plus standard care compared with standard care for treating eczema in children: A randomised, controlled, observer-blind, pragmatic trial (CLOTHES Trial)
Source: PLoS Med. 2017 Apr 11;14(4):e1002280. doi: 10.1371/journal.pmed.1002280 (PMC5388469; doi:10.1371/journal.pmed.1002280)
Supplement: S8 Table — (DOCX) [file pmed.1002280.s013.docx]

S8 Table: Incremental Cost-Effectiveness Analyses Results for base case and sensitivity analysis testing an alternative approach to costing silk garments

| **Analysis** | **N per arm (Int; Cont)** | **Adjusted Incremental healthcare costs (including garments) (CI)** | **Adjusted Incremental outcome (CI)** | **ICER for NHS perspective** |
| --- | --- | --- | --- | --- |
| **Base case:**  **PCA Cost / ADQoL** | 134; 139 | 364.94  (217.47, 512.42) | 0.0064 (-0.0004, 0.0133) | £56,811 per QALY |
| **Sensitivity analysis:**  **Tariff cost / ADQoL** | 134; 139 | 346.46  (199.08, 493.84) | 0.0064 (-0.0004, 0.0133) | £53,989 per QALY |
