# Supplementary material for: Silk garments plus standard care compared with standard care for treating eczema in children: A randomised, controlled, observer-blind, pragmatic trial (CLOTHES Trial)
Source: PLoS Med. 2017 Apr 11;14(4):e1002280. doi: 10.1371/journal.pmed.1002280 (PMC5388469; doi:10.1371/journal.pmed.1002280)
Supplement: S1 Protocol — (PDF) [file pmed.1002280.s014.pdf]

Randomised controlled trial of silk therapeutic clothing for the long-term management of eczema in children

Final 3.0
11 February 2014

Short title: **CLOTHES Trial – Clothing for the relief of Eczema Symptoms**

Acronym: CLOTHES

ISRCTN: 77261365

NRES reference: **13/EM/0255**

Trial Sponsor: University of Nottingham

Sponsor reference: **13067**

Funding Source: NIHR Health Technology Assessment
Ref 11/65/01

This protocol is confidential and the property of the University of Nottingham. No part of it may be transmitted, reproduced, published, or used by others persons without prior written authorisation from the University of Nottingham

TRIAL / STUDY PERSONNEL AND CONTACT DETAILS

**Sponsor:**<br>Contact name	University of Nottingham<br>Mr Paul Cartledge<br>Head of Research Grants and Contracts<br>Research Innovation Services<br>King's Meadow Campus<br>Lenton Lane<br>Nottingham<br>NG7 2NR
**Chief investigator:**	Kim Thomas PhD<br>Professor of Applied Dermatology Research<br>Centre of Evidence Based Dermatology<br>University of Nottingham<br>Nottingham NG7 2UH<br>Phone: 0115 8468632<br>Email: kim.thomas@nottingham.ac.uk
**Medical Expert:**	Hywel Williams MSc PhD FRCP<br>Professor of Dermato-Epidemiology<br>Centre of Evidence Based Dermatology<br>University of Nottingham<br>Nottingham University Hospitals NHS Trust Queen's Medical Centre<br>Nottingham NG7 2UH<br>Tel: 0115 8231048<br>Email: hywel.williams@nottingham.ac.uk
**Qualitative Lead:**	Dr Fiona Cowdell<br>Senior Research Fellow<br>Faculty of Health and Social Care<br>Room 204, Dearne Building<br>University of Hull<br>Hull HU6 7RX<br>Tel: 01482 463 362<br>E-mail: f.cowdell@hull.ac.uk
**Co-investigators:**	Professor Tara Dean<br>Director of Research<br>School of Health Sciences and Social Work<br>University of Portsmouth<br>James Watson Building<br>2 King Richard Road<br>Portsmouth PO1 2FR<br>Tel: 02392 844405<br>E-mail: tara.dean@port.ac.uk<br><br>Dr Tracey Sach<br>Reader in Health Economics<br>Norwich Medical School

This protocol is confidential and the property of the University of Nottingham. No part of it may be transmitted, reproduced, published, or used by others persons without prior written authorisation from the University of Nottingham

University of East Anglia
Norwich Research Park
Norwich NE4 7TJ
Tel: 01603 592022
E-mail: T.Sach@uea.ac.uk

Mrs Sandra Lawton
Nurse Consultant in Dermatology
Queens Medical Centre
Nottingham University Hospitals NHS Trust
Nottingham NG7 2UH
Tel: 0115 924 9924 x64737
E-mail: Sandra.lawton@nuh.nhs.uk

Dr Ian Pollock
Consultant Paediatrician
Paediatrics
Barnet and Chase Farm Hospitals NHS Trust
Chase Farm Hospital
The Ridgeway
Enfield
Middlesex EN2 8JL
Tel: 020 8375 1900
E-mail: ian.pollock@bcf.nhs.uk

Dr Nigel Burrows
Consultant Dermatologist
Department of Dermatology, Box 46
Addenbrookes NHS Trust
Hills Road
Cambridge CB2 0QQ
Tel: 01223 216 459
E-mail: nigel.burrows@addenbrookes.nhs.uk

Dr Jonathan Batchelor
Dermatologist
Centre for Evidence Based Dermatology
University of Nottingham
Kings Meadow Campus
Lenton Lane
Nottingham NG7 2NR
Tel: 0115 951 5151 x66947
E-mail: jonathan.batchelor@nottingham.ac.uk

This protocol is confidential and the property of the University of Nottingham. No part of it may be transmitted, reproduced, published, or used by others persons without prior written authorisation from the University of Nottingham

Dr Sara Brown
Honorary Consultant Dermatologist
Wellcome Trust Intermediate Clinical Fellow & Clinical Senior Lecturer
Department of Dermatology
Ninewells Hospital
Dundee DD1 9SY
Tel: 01382 660111 x 36244
E-mail: sarabrown1@nhs.net

Prof Alan Montgomery
Professor of Medical Statistics and Clinical Trials
Nottingham Clinical Trials Unit
University of Nottingham
Nottingham Health Science Partners
Nottingham University Hospitals NHS Trust Queen's Medical Centre
Nottingham NG7 2UH
Tel: 0115 884 4953
E-mail: alan.montgomery@nottingham.ac.uk

Trial Statistician: Prof Alan Montgomery (as above)

Trial Coordinating Centre:

Nottingham Clinical Trials Unit
University of Nottingham
Nottingham Health Science Partners
Nottingham University Hospitals NHS Trust Queen's Medical Centre
Nottingham NG7 2UH
Tel: 0115 8844919.

This protocol is confidential and the property of the University of Nottingham. No part of it may be transmitted, reproduced, published, or used by others persons without prior written authorisation from the University of Nottingham

SYNOPSIS

Title	Randomised controlled trial of silk therapeutic clothing for the long-term management of eczema in children
Acronym	CLOTHES
Short title	Clothing for the Relief of Eczema Symptoms
Chief Investigator	Dr Kim Thomas
Objectives	To assess whether silk therapeutic clothing, when used in addition to standard eczema care, reduces eczema severity in children over a period of six months.
Trial Configuration	Observer-blind, parallel group randomised controlled trial with optional qualitative component
Setting	Recruitment into this trial will be based in secondary care hospitals. GP practices and other local hospitals within travelling distance of the recruiting hospital will be approached to act as Patient Identification Centres (PICS).
Sample size estimate	Three hundred participants provides 90% power, at the 5% significance (two-tailed) to detect a 3-point difference between the groups in mean EASI scores over the first 6 months of the study; assuming a SD of 13, a correlation between EASI scores at different time points of 0.6 and loss to follow up of 10%.
Number of participants	300 children aged 1 to 15 years
Eligibility criteria	Participants aged 1 to 15 years with moderate to severe eczema will be eligible to participate.
Description of interventions	The medical device under investigation is a knitted, sericin-free silk therapeutic garment with a CE mark for use in eczema. Patients will be randomised to standard care alone or standard care plus therapeutic clothing.
Duration of study	Overall duration of the study is anticipated to be approximately 36 months and is planned to start in Q2 2013. Each participant will participate in the trial for 8 months with an optional interview or focus group after end of participation.
Randomisation and blinding	The randomisation schedule is based on a computer generated pseudo-random code using random permuted blocks of randomly varying size and is stratified by site and age. Research nurses will assess eczema severity (primary outcome) and will remain blinded throughout the trial.

This protocol is confidential and the property of the University of Nottingham. No part of it may be transmitted, reproduced, published, or used by others persons without prior written authorisation from the University of Nottingham

Outcome measures	Primary outcome will be eczema severity measured by the objective Eczema Area and Severity Index (EASI) at baseline 2, 4 and 6 months.<br><br>Qualitative outcome measures will include participant's and parent's experience of silk garments use and the views of prescribers / commissioners on prescribing silk garments.
Statistical methods	All analyses will be carried out using Stata 11.2 or MLwiN. This trial will be analysed as intention-to-treat (ITT) at 6 months. The primary analysis of EASI will be performed using a multilevel model framework with observations at 2, 4 and 6 months, nested with participants and including baseline EASI and the stratification factors (site and age group) as covariates.<br><br>Qualitative data will be analysed thematically.

This protocol is confidential and the property of the University of Nottingham. No part of it may be transmitted, reproduced, published, or used by others persons without prior written authorisation from the University of Nottingham

ABBREVIATIONS

ADQoL	Atopic Dermatitis Quality of life preference based index
AE	Adverse Event
ANCOVA	Analysis of Covariance
CI	Chief Investigator
CRF	Case Report Form
CTU	Clinical Trials Unit
DFI	Dermatitis Family Impact Questionnaire
EASI	Eczema Area and Severity Index
EQ-5D-3L	EuroQol 5 Dimension 3 level questionnaire
FLG	Gene Encoding Filaggrin
GCP	Good Clinical Practice
HTA	Health Technology Assessment
ICF	Informed Consent Form
IGA	Investigator Global Assessment
ITT	Intention To Treat
MLM	Multilevel model
NESS	Nottingham Eczema Severity Scale
NHS	National Health Service
NIHR	National Institute of Health Research
PGA	Patient Global Assessment
P/GIS	Parent / Guardian Information Sheet
PI	Principal Investigator at a local centre
PIS	Participant Information Sheet
POEM	Patient Oriented Eczema Measure
RCT	Randomised Control Trial
REC	Research Ethics Committee
R&D	Research and Development department
SAE	Serious Adverse Event
SAP	Statistical Analysis Plan
SOP	Standard Operating Procedure
TIS	Three Item Severity Scale
TMG	Trial Management Group
TSC	Trial Steering Committee

This protocol is confidential and the property of the University of Nottingham. No part of it may be transmitted, reproduced, published, or used by others persons without prior written authorisation from the University of Nottingham

TABLE OF CONTENTS

SYNOPSIS **5**

ABBREVIATIONS **7**

TRIAL / STUDY BACKGROUND INFORMATION AND RATIONALE **10**

TRIAL / STUDY OBJECTIVES AND PURPOSE **11**
PURPOSE 11
PRIMARY OBJECTIVE 11
SECONDARY OBJECTIVE 11

TRIAL / STUDY DESIGN **11**
TRIAL / STUDY CONFIGURATION 11
FIGURE 2 - FLOWCHART **ERROR! BOOKMARK NOT DEFINED.**
Primary endpoint 13
Secondary endpoints 13
Safety endpoints 14
Tertiary endpoints: 14
Stopping rules and discontinuation 14
TABLE 1 - PILOT STOP/GO CRITERIA 15
RANDOMISATION AND BLINDING 15
Maintenance of randomisation codes and procedures for breaking code 16
TRIAL MANAGEMENT 16
DURATION OF THE TRIAL / STUDY AND PARTICIPANT INVOLVEMENT 17
End of the Trial 17
SELECTION AND WITHDRAWAL OF PARTICIPANTS 17
Recruitment 17
Eligibility criteria 18
Inclusion criteria 18
Exclusion criteria 18
ADDITIONAL GUIDANCE ON ELIGIBILITY CRITERIA 18
Expected duration of participation 19
Removal of participants from therapy or assessments 19
Informed consent 20

TRIAL INTERVENTION AND REGIMEN **21**
Interventions: 21
TABLE 2 - STUDY ASSESSMENTS 22
DATA COLLECTION METHODS 23
Criteria for terminating trial 24
OPTIONAL QUALITATIVE INTERVIEW/ FOCUS GROUP METHODS 24
FILAGRIN GENETICS ANALYSES 27

STATISTICS **28**
Methods 28
Sample size and justification 28
Procedures for missing, unused and spurious data 28

COST-EFFECTIVENESS ANALYSIS **30**
OBJECTIVE: 30

This protocol is confidential and the property of the University of Nottingham. No part of it may be transmitted, reproduced, published, or used by others persons without prior written authorisation from the University of Nottingham

ADVERSE EVENTS **30**
TRIAL INTERVENTION-RELATED SAES 30

ETHICAL AND REGULATORY ASPECTS **31**
ETHICS COMMITTEE AND REGULATORY APPROVALS 31
INFORMED CONSENT AND PARTICIPANT INFORMATION 32
RECORDS 33
Case Report Forms and patient diaries 33
Sample Labelling 33
Source documents 33
Direct access to source data / documents 33
DATA PROTECTION 33

QUALITY ASSURANCE & AUDIT **34**
INSURANCE AND INDEMNITY 34
TRIAL CONDUCT 34
TRIAL DATA 34
RECORD RETENTION AND ARCHIVING 34
DISCONTINUATION OF THE TRIAL BY THE SPONSOR 35
STATEMENT OF CONFIDENTIALITY 35

PUBLICATION AND DISSEMINATION POLICY **35**

USER AND PUBLIC INVOLVEMENT **35**

STUDY FINANCES **35**
Funding source 35
Participant stipends and payments 35

SIGNATURE PAGES **36**

REFERENCES **37**

This protocol is confidential and the property of the University of Nottingham. No part of it may be transmitted, reproduced, published, or used by others persons without prior written authorisation from the University of Nottingham

TRIAL / STUDY BACKGROUND INFORMATION AND RATIONALE

Study Background

Eczema is a chronic, inflammatory skin condition that is common in childhood (Williams, Stewart et al. 2008). Whilst most cases of eczema can be successfully treated with topical medications, many parents express concern at using these preparations and are keen to explore non-pharmacological treatment options.

It has long been understood that clothing can cause irritation to the skin, and current guidelines recommend the use of loose cotton clothing, and the avoidance of wool and other rough fibres next to the skin http://www.nhs.uk/Conditions/Eczema-(atopic)/Pages/Treatment.aspx). In response to this need, new clothing products have become available in recent years, and these are now marketed as having beneficial effects in the treatment of eczema. The therapeutic silk garments included in this study are available on prescription through the NHS, but the trial evidence supporting their use is currently limited(Vlachou, Thomas et al. 2009).

The effectiveness of these therapeutic silk garments is lodged as a treatment uncertainty in the UK Database of Uncertainties about the Effectiveness of Treatments (DUETs).

Loss of function mutations in the gene encoding filaggrin (*FLG*) affect skin barrier function and are a strong and significant risk factor in eczema. These mutations are present in approximately 9% of the UK population and because of their role in skin barrier formation they may affect an individual's response to silk clothing as a treatment for eczema.

Existing research

The 2000 HTA systematic review of eczema treatments(Hoare, Li Wan Po et al. 2000) found no RCTs looking at therapeutic clothing for the treatment of eczema, although there was some evidence to suggest that smoother fibres were less aggravating to eczematous skin(Diepgen, Salzer et al. 1995). This review has recently been updated and has revealed six small RCTs looking at therapeutic clothing for the treatment of eczema; two using silver coated textiles(Gauger, Fischer et al. 2006),(Juenger, Ladwig et al. 2006) one using MEDIELE(Ozawa 2008), one using Ethylene Vinyl Alcohol (EVOH) fibre(Yokoyama, Kimata et al. 2009) and two RCTs using DermaSilk™ (Koller, Halmerbauer et al. 2007; Stinco, Piccirillo et al. 2008),. These silk clothing trials were too small (22 and 30 participants respectively) and of limited duration (12 and 4 weeks respectively) to inform clinical practice.

On the basis of the limited trial data to-date, it is not possible to establish whether or not silk clothing provides measurable benefits for patients with atopic eczema. Despite the limited trial evidence, these garments are currently available on prescription in the UK and their popularity amongst patients is growing. The purpose of the optional qualitative element is to elicit detailed information from parent / guardians and children about factors that influence the use of silk clothing and to understand prescribers / commissioners view on the use of silk garments. Qualitative data will be aligned with quantitative findings to provide a rounded understanding of garment usage including barriers and enablers.

This protocol is confidential and the property of the University of Nottingham. No part of it may be transmitted, reproduced, published, or used by others persons without prior written authorisation from the University of Nottingham

TRIAL / STUDY OBJECTIVES AND PURPOSE

PURPOSE

The purpose of this study is to establish whether silk therapeutic clothing is effective in the long-term management of eczema in children.

PRIMARY OBJECTIVE

To assess whether silk therapeutic clothing, when used in addition to standard eczema care, reduces eczema severity in children over a period of six months.

SECONDARY OBJECTIVE

1. To estimate the within trial cost-effectiveness of silk therapeutic clothing with standard care, compared to standard care alone, from an NHS and a family perspective.
2. To explore parent/ guardian and child views on and experiences of using silk garments and factors that might influence the use of these garments in everyday life.
3. To examine prescribers /commissioners views of the use of silk garments.

TRIAL / STUDY DESIGN

TRIAL / STUDY CONFIGURATION

Three hundred children with eczema (atopic dermatitis) will be randomised into a study comprising an independent, observer-blind, parallel group randomised controlled trial (RCT) to evaluate the addition of silk therapeutic clothing (a CE marked medical device), compared to standard eczema care (Figure 1), followed by an open follow-up period.

Figure 1: Trial Design

Length of study		0 to 6 months		7 to 8 months	
Intervention Group	Randomisation	Standard care + silk clothing	End of RCT	Standard care + silk clothing	Optional Interview or focus group
Control Group		Standard care only		Standard care + silk clothing	

Children will be randomised to receive standard care plus silk therapeutic clothing (three sets of long sleeved tops and leggings, or bodysuits for infants), or standard care alone. Patients randomised to therapeutic clothing will be further randomised to have one of two different types (brands) available on prescription - Dermasilk™ and Dreamskin™. Patients will be unaware of the brand of clothing they will receive. The primary outcome for the RCT (eczema severity measured by EASI), will be assessed at baseline, two, four and six months by research nurses blinded to allocation status.

This protocol is confidential and the property of the University of Nottingham. No part of it may be transmitted, reproduced, published, or used by others persons without prior written authorisation from the University of Nottingham

In order to provide additional information about the clothing and potentially encourage continued participation in the standard care group, participants will receive the clothing (randomly assigned to one of two different types (brands) available on prescription - Dermasilk™ and Dreamskin™), on completion of the randomised controlled part of the study at six months. Patients will be unaware of the brand of clothing they will receive. At eight months from randomisation both groups will be asked to complete a questionnaire recording eczema symptoms, and satisfaction with the clothing (including adherence, acceptability and durability).

All patients (in specified sites) will also be given the choice to participate in an optional interview or focus group after eight months post randomisation. Data from this qualitative component to the trial will add an additional person-centred account to the RCT data: For example if no overall benefits are found in the RCT the qualitative work may illuminate if the garments have been worn and, if not, the reasons why this is the case. Equally if an overall benefit is found in the RCT the qualitative study may clarify if this is related to the RCT environment (such as increased concordance due to attention given to participants) or whether benefits could be further enhanced if findings from the focus groups are acted upon.

Prescribers and commissioners

Prescribers will be recruited throughout the study via relevant professional groups such as the British Association of Dermatologists and the British Dermatological Nursing Group. Commissioners will be recruited via professional networks.

To determine whether there is an effect of mutations of the gene encoding for filaggrin (an abnormality associated with impaired skin barrier function) saliva samples will be taken from participants where separate informed consent has been obtained at their first visit.

An internal pilot RCT will be conducted over the first six months of trial recruitment in order to assess ability to recruit, adherence with the clothing and retention in the trial.

This protocol is confidential and the property of the University of Nottingham. No part of it may be transmitted, reproduced, published, or used by others persons without prior written authorisation from the University of Nottingham

Figure 2: Flowchart

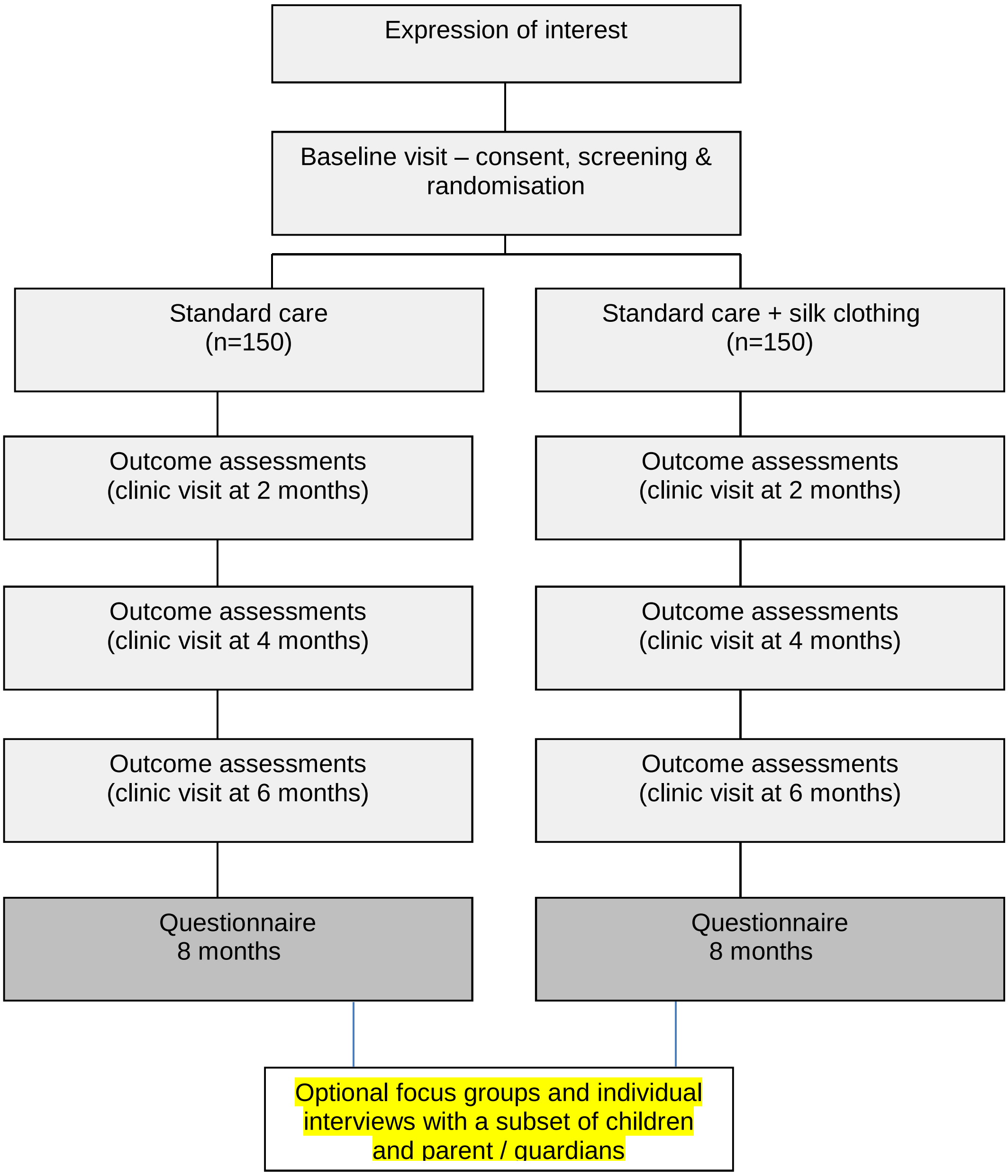

Primary endpoint

The primary end point is eczema severity measured by the objective Eczema Area and Severity Index (EASI)(Barbier, Paul et al. 2004) at baseline 2, 4 and 6 months. Assessments will be made by research nurses who have been trained in using the EASI tool and who are blinded to group allocation. The same research nurse will assess the skin at each time point for each participant in order to minimise inter-observer variability.

Secondary endpoints

a) Global assessment of the eczema, assessed by research nurses (Investigator Global Assessment: IGA) and by participants (Participant Global Assessment: PGA) at baseline, 2, 4 and 6 months.

This protocol is confidential and the property of the University of Nottingham. No part of it may be transmitted, reproduced, published, or used by others persons without prior written authorisation from the University of Nottingham

b) Three Item Severity scale (TIS) at baseline, 2, 4 and 6 months, assessed by the research nurses and used to assess eczema severity.

c) Use of topical treatments: use and potency of topical steroids and topical calcineurin inhibitors, use of emollients and frequency of wet / dry wrapping throughout the trial.

d) Self reported eczema symptoms using the Patient Oriented Eczema measure (POEM)(Charman, Venn et al. 2004)

e) Health Related Quality of Life at baseline and 6 months: Dermatitis Family Impact (DFI)(Lawson, Lewis-Jones et al. 1998); will assess the impact of the child's health on the whole family, EQ-5D-3L will provide a utility measure for the main carer; and age-appropriate utility measures (Atopic Dermatitis Quality of life preference based index ADQoL)) will be used to provide an eczema-specific utility score(Stevens, Brazier et al. 2005), and a general health utility score for the child (CHU-9D)(Stevens 2012)

f) Durability of the garments, adherence and acceptability of use (as assessed by children and parents/carers).

g) Cost-effectiveness and cost utility.

The number of children with mutations on the gene encoding for filaggrin will also be reported and used to inform a planned sub-group analysis, to test the hypothesis that children with at least one mutation on the gene encoding for filaggrin are more likely to respond favourably to the therapeutic clothing if it compensates for loss of barrier function. Specific consent for this testing will be obtained.

Safety endpoints

a) Number of skin infections – defined as patient-reported skin infections that require antibiotic or antiviral treatment.

Given that it is unlikely that silk clothing will result in any adverse device effects other than potential changes in the number of skin infections, only serious adverse events will be collected.

Tertiary endpoints:

Additional exploratory analysis will be conducted based on eczema severity scores in areas covered by the clothing (body and limbs) compared to areas uncovered by the clothing (head and neck), in order to test the theory that gaining eczema control in one site may reduce a patient's overall immunological response, and therefore disease activity at distant sites.

Whilst it is assumed that the different brands of clothing be similar, the effects in each group may be investigated, if appropriate.

The examination of all tertiary end points will be considered exploratory.

Further details about the statistical analyses will be provided in the Statistical Analysis Plan which will be finalised prior to database lock and unblinding of the study.

Stopping rules and discontinuation

An internal pilot RCT will be conducted over the first six months of trial recruitment. Progress with recruitment and retention will be monitored monthly by the Trial Management Group (TMG). If progress is below target, strategies will be implemented to improve progress.

This protocol is confidential and the property of the University of Nottingham. No part of it may be transmitted, reproduced, published, or used by others persons without prior written authorisation from the University of Nottingham

Table 1 pre-specifies the stop/go criteria to be assessed by the Trial Steering Committee at 6 months (target recruitment = 75 participants within six months of the first participant being randomised).

Table 1 - Pilot stop/go criteria

Criteria to be assessed at 6 months of recruitment	Proposed action
≥90% of target recruitment & retention	Continue with main trial as planned
70% to 89% of target recruitment & retention	Continue with main trial. Implement strategies for improvement.
50% - 69% of target recruitment & retention	Urgent measures required, discuss plans with Trial Steering Committee and NIHR HTA
<50% of target recruitment & retention	Stop trial unless good reason for delay and rectifiable solution can be readily implemented

Adherence in wearing the clothing will be assessed regularly by the TMG in the intervention group only, and will act as a trigger for concern if participants report using the clothing less than 50% of the time.

The Sponsor reserves the right to discontinue this study at any time for failure to meet expected enrolment goals, for safety or any other administrative reasons. The Sponsor shall take advice from the Trial Steering Committee as appropriate in making this decision.

RANDOMISATION AND BLINDING

The randomisation schedule is based on a computer generated pseudo-random code using random permuted blocks of randomly varying size, created by the Nottingham Clinical Trials Unit (CTU) in accordance with their standard operating procedure (SOP) and held on a secure University of Nottingham server. The randomisation is stratified by recruiting hospital and by child's age - <2years; 2 to 5 years; and >5 years.

Access to the sequence will be confined to the data management team and administrators at the Nottingham CTU who will be responsible for distribution of the clothing. Investigators and Research Nurses will access the randomisation website by means of a remote, internet-based randomisation system developed and maintained by the Nottingham CTU. The sequence of treatment allocations will be concealed until interventions have all been assigned and recruitment, data collection, and all other trial-related assessments are complete.

Participants will be informed of their treatment allocation by letter from the co-ordinating centre. Those allocated to receive the therapeutic clothing will receive their first supplies at this time. Participants will be randomly allocated to one of the therapeutic clothing brands available on prescription in the UK as they enter the trial. They will not be aware of which brand of clothing they have received. Packaging, labelling and distribution of clothing will be performed by staff at the co-ordinating centre and sent to participants by post. Sizes will be determined by collection of the participant's height at randomisation

Whilst it will not be possible to blind participants to their treatment allocation, efforts will be made to minimise expectation bias by emphasising in the trial literature that the evidence supporting the use of therapeutic clothing for eczema is currently limited, and that we do not

This protocol is confidential and the property of the University of Nottingham. No part of it may be transmitted, reproduced, published, or used by others persons without prior written authorisation from the University of Nottingham

yet know if this clothing offers any benefit over standard care. The study literature will also avoid the use of terms such as “specialist” or “therapeutic” clothing for the same reason.

Where possible research nurses will remain blinded throughout the trial: participants will be reminded in the study literature and in their clinic appointment letters not to wear the clothing when they attend the clinic, or to mention the clothing in any way when talking to the research nurses. All questions relating to the acceptability and use of the clothing will be completed by either postal or on-line questionnaire (according to patient preference), and telephone contact with participants will be made by trial staff from the co-ordinating centre whenever possible. If the research nurses become unblinded, this will be recorded and used to inform a sensitivity analysis.

	**Blinding status**	**Comments**
**Participants**	Not Blinded	Not possible to blind participants, efforts will be made to minimise expectation bias.
**Research nurses and PI**	Blinded	Participants will be reminded in their clinic appointment letters not to wear the clothing when they attend the clinic, or to mention the clothing in any way when talking to the research nurses.
**Trial staff at Nottingham CTU**	Not blinded	Will be the main point of contact for participants wishing to contact the research team, will package and post the clothing to the participants according to the randomisation schedule, and will provide general advice.
**Statistician**	Blinded	Statistician will finalise the analysis plan prior to revealing the treatment codes.

Maintenance of randomisation codes and procedures for breaking code

Only the trial team and the data manager at the Nottingham CTU will be aware of the allocation to intervention or control group. Since the intervention is clothing, no special arrangements are necessary for breaking of the randomisation code.

TRIAL MANAGEMENT

The trial is funded by the NHS Health Technology Assessment Programme. It is sponsored by the University of Nottingham, and will be managed and co-ordinated from the Centre of Evidence Based Dermatology in Nottingham and the Nottingham Clinical Trials Unit.

The Trial Steering Committee (TSC) will meet at least once a year and will provide overall supervision of the trial on behalf of the trial sponsor.

The Trial Management Group (TMG) will meet more frequently and will be responsible for the day-to-day management of the trial. Members of the TMG will report to the TSC at their meetings.

Due to the low risk of the clothing, no data monitoring committee will be required.

This protocol is confidential and the property of the University of Nottingham. No part of it may be transmitted, reproduced, published, or used by others persons without prior written authorisation from the University of Nottingham

The Chief Investigator has overall responsibility for the study and shall oversee all study management.

The data custodian will be the Chief Investigator.

DURATION OF THE TRIAL / STUDY AND PARTICIPANT INVOLVEMENT

The overall duration of the study is anticipated to be approximately 36 months. It is planned that recruitment will take up to 18 months. Target recruitment for the pilot phase of the trial is 75 participants enrolled over the first 6 months of recruitment.

Each participant will participate in the trial for 8 months (treatment period) and her/his participation will commence upon signing the consent form. Participants will be randomised to intervention or control groups for the first 6 months. Participation in the RCT will end after 6 months, at which point participants in the control group (standard care) will be given the therapeutic clothing and will be followed up until 8 months post randomisation. Participants allocated to the intervention group will continue with their randomised intervention until the garments are no longer usable. Further supplies of clothing will not be given to these patients during the follow-up period from 6 to 8 months post-randomisation.

Participation in the qualitative component will be a maximum of 2.5 additional hours after completing their final 8 month contact.

End of the Trial

The end of the RCT will be receipt of the last questionnaire of the last participant.

The end of the qualitative study will occur when the final focus groups and interviews are completed for the sub-set of patients who have volunteered to participate in the qualitative component and the cohort of commissioners and clinicians have been interviewed..

SELECTION AND WITHDRAWAL OF PARTICIPANTS

Recruitment

Recruitment into this trial will be based in six secondary care hospitals (with further contingency centres as required). GP practices and other local hospitals in the surrounding area will be approached to act as Patient Identification Centres (PICS).

Potential participants will also be identified through direct local advertising (including but not limited to e-mail distribution lists, radio and TV interviews, websites and articles). If other potential sources for recruitment become apparent during the trial, these will also be included. A dedicated website will be available for the purpose of this trial.

If identified through secondary or primary care the initial approach will be from a member of the patient's usual care team (which may include the investigator), and information about the trial will be on display in the relevant clinical areas. The initial approach may be made by invitation letter on in person at a clinic.

The investigator or their nominee, e.g. from the research team, will inform the participant and their parent/legal guardian of all aspects pertaining to participation in the study.

If needed, the usual hospital interpreter and translator services will be available to assist with discussion of the trial, the participant information sheets, and consent forms, but the consent forms and information sheets will not be available printed in other languages.

This protocol is confidential and the property of the University of Nottingham. No part of it may be transmitted, reproduced, published, or used by others persons without prior written authorisation from the University of Nottingham

It will be explained to the potential participant and their parent/legal guardian that entry into the trial is entirely voluntary and that their treatment and care will not be affected by their decision. It will also be explained that they can withdraw at any time.

Eligibility criteria

Inclusion criteria

- Children aged 1 to 15 years at baseline.
- Diagnosis of moderate or severe eczema (atopic dermatitis). Presence of eczema will be confirmed using the UK Diagnostic Criteria for Atopic Eczema(Williams, Burney et al. 1994) and eczema severity judged using the Nottingham Eczema Severity Scale (NESS)(Emerson 2000)
- Resident within travelling distance of a recruiting centre.
- Children with at least one patch of eczema on the trunk or limbs
- Parent/legal guardian able to give informed consent

Exclusion criteria

- Children who have taken systemic medication (including light therapy) or oral steroids for eczema within the previous three months.
- Children who have started a new treatment regimen within the last month.
- Children who have used wet/dry wraps ≥5 times in the last month.
- Children who are currently using silk clothing for their eczema and are unwilling to stop using the clothing during the trial.
- Children who are currently taking part in another clinical trial.
- Children who have expressed a wish not to take part in the trial.

Additional guidance on eligibility criteria

'Children who have started a new treatment regimen within the last month' are excluded from taking part in the trial. This should be interpreted as a new treatment regimen for eczema, or if they have started or are currently using any treatment that may affect eczema

A change in treatment regimen for eczema would include a change in strength of topical corticosteroids or addition of calcineurin inhibitors, and therefore the child would be excluded. However, changes to the same strength of steroid, and changes to emollients are acceptable. Changes in diet to help control eczema are also acceptable, as well as the use of antibiotics, in the previous 3 months, for an infection due to the child's eczema.

Any new or existing treatment for other conditions that require anti-inflammatory medications e.g. oral steroids for asthma, arthritis or inflammatory bowel disease, would also fall under this exclusion criteria and therefore the child would not be eligible to take part. However, steroid inhalers for asthma do NOT fall under this exclusion criteria.

The use of oral antihistamines, NSAIDs and steroid inhalers within the past month are permitted. If any of the following treatments have been used within the last month, for any condition, the child would be excluded: methotrexate, ciclosporin, prenisolone, azathioprine, light therapy, mycophenolate mofetil.

Only one child will be enrolled per family. The choice as to which child becomes involved will be made by the parents and children involved, taking into account the eligibility criteria above.

This protocol is confidential and the property of the University of Nottingham. No part of it may be transmitted, reproduced, published, or used by others persons without prior written authorisation from the University of Nottingham

Included in the 8 month letter to parents and children, parents/guardians will be sent an invite to the qualitative component of the study, as well as parent and age-appropriate child information sheets. Patients and their families may choose to contact the Qualitative Lead to express interest in the interviews/focus groups.

Children and Parents/ guardians will be aware that not choosing to take part in the qualitative interviews/ focus groups will in no way effect their participation in the RCT or their future care.

Prescribers and commissioner will be identified via existing dermatology networks at the beginning of the study and brief telephone interviews arranged.

Eligibility Criteria for the Optional Qualitative Component:

Inclusion criteria
Children

- Participants in the main CLOTHES trial.
- Parent / guardian able to give informed consent.
- Child able to give assent.

Parent / guardians

- Parent / guardian of child in the main CLOTHES trial
- Able to give informed consent.

Prescribers / commissioners

- Active involvement in prescribing or commissioning eczema treatments, including but not limited to the provision of silk garments for childhood eczema.

Exclusion criteria
Children

- Unwilling to take part
- Unable to converse in English language

Parent / guardians

- Unwilling to take part
- Unable to converse in English language

Prescribers / commissioners
Unwilling to take part

Expected duration of participation

Study participants will be enrolled in the study for 8 months.

Study participants (See Qualitative Component Research Methods, page 24 for maximum numbers) who opt to do the additional qualitative interview/focus group will extend their participation for another 2.5 hours beyond the 8 months of the RCT and RCT follow-up.

Removal of participants from therapy or assessments

Participants (child or parent/ legal guardian) may be withdrawn from the trial at any time. They will be made aware (through the participant information sheets) that withdrawing from the trial will not affect their future care, but that the data already collected cannot be erased and will still be used in the final analysis. The primary reasons for discontinuation/withdrawal will be recorded.

This protocol is confidential and the property of the University of Nottingham. No part of it may be transmitted, reproduced, published, or used by others persons without prior written authorisation from the University of Nottingham

Participants who withdraw after randomisation will not be replaced.

Informed consent

RCT

All parents/legal guardians will provide written informed consent for their child to take part in the trial. The Informed Consent Form will be signed and dated by the parent/legal guardian before they enter the trial. For children wishing to do so, assent will be given on the same form, but this will not be mandatory. In the event of any conflict between the parent's and child's wishes, the child WILL NOT enter the study. The Investigator will explain the details of the trial and provide age appropriate Participant Information Sheets, ensuring that the participant and/or parent/legal guardian has sufficient time to consider participating or not. The Investigator will answer any questions that the child and/or parent/legal guardian has concerning study participation.

Separate informed consent will be obtained for the provision of a saliva sample (for genetic testing), and parents / legal guardians will be offered the possibility of opting out of this part of the study if they wish. No samples will be taken until informed consent has been gained. In line with current guidance patients will not receive their filaggrin test results (Fabsitz, McGuire et al. 2010)

Informed consent will be collected from each parent/legal guardian before they undergo any interventions (including physical examination) related to the study. One copy of this will be kept by the parent/legal guardian, one will be kept by the Investigator, and a third will retained in the child's notes.

Should there be any subsequent amendment to the final protocol that might substantially affect a participant's participation in the trial, continuing consent will be obtained using an amended Consent form, which will be signed by the parent/legal guardian.

Qualitative Interview/ Focus Group

The Investigator facilitating the focus group or interview will obtain consent from each participant at the onset of the interview/ focus group. Parents/guardians and children will have been provided with detailed, age appropriate Participant Information Sheets in the 8 month RCT study mailing, and will be given them again at the onset of the interview/focus group. Prior to the interview or focus group, they will have the opportunity to ask questions about the qualitative study. The Investigator will explain the details of the qualitative component and answer any questions that the participant has.

Parent / guardians will provide written consent prior to participation of themselves and their child. Children will have an opportunity to sign assent forms if appropriate for their age and ability to understand the study. In the event of any conflict between the parent / guardian and child the child will not enter the study.

Consent will be obtained on one occasion only. One copy will be kept by the participant and one will be kept by the Investigator,

Verbal consent will be taken, and noted, at the beginning of interviews with prescribers/commissioners.

This protocol is confidential and the property of the University of Nottingham. No part of it may be transmitted, reproduced, published, or used by others persons without prior written authorisation from the University of Nottingham

TRIAL INTERVENTION AND REGIMEN

Interventions:

Silk clothing:

The medical device under investigation is a knitted, sericin-free silk therapeutic garment with a CE mark for use in eczema. Sericin is a protein that coats the outside of silk fibres and has the potential to cause allergic reactions. Medical grade silk (such as silk used for stitches during operations), has the sericin removed for this reason.

The specific products being used are Dreamskin™ and Dermasilk™. The silk clothing will be worn at night, and when possible during the day. Participants will receive three sets of garments (long-sleeved vest and leggings, or body suits and leggings depending on the age of the child). This will give sufficient garments to allow for washing between uses. Washing instructions will be provided in line with manufacturers recommendations. Adherence in wearing the garments will be collected using sticker/tick charts as an aide memoir and weekly questionnaires.

The clothing will have product labels removed and be supplied using trial-specific packaging prior to distribution from the co-ordinating centre. Clothing will be replaced as required during the six-month RCT (e.g. because they are worn out or because the child has grown). Once the randomised part of the trial is complete at six months, garments will NOT be replaced.

Participants will be asked about their use of clothing and willingness of GPs to prescribe them at 8 months. It will be explained to participants at this stage that it is not possible for the trial team to recommend use of the garments, or to insist that their GPs prescribe them.

Participants allocated to the therapeutic clothing will continue to use their standard eczema care (including emollients, topical corticosteroids and topical calcineurin inhibitors).

Standard care:

All participants (active and control groups) will continue with their standard eczema care in line with NICE guidance (NICE December 2007). No efforts will be made to intervene or change a child's standard eczema care unless the research nurse thinks that the skin may be infected. If research nurses suspect infection they will recommend that the patients contact their normal medical team (GP, Nurse, dermatologist) as appropriate.

Standard advice about what clothing to use for a child with eczema will be provided (avoid wool, and wear cool loose clothing – especially cotton and linen), but specific products will not be recommended.

If a child is currently using "specialist" cotton clothing (e.g. special sleep suits with built-in mittens), the use of these garments will be recorded, but will not be grounds for exclusion. However, participants in the control group will be asked to refrain from using prescription clothing (including silk clothing and synthetic garments used for wet wrapping) during the trial.

This protocol is confidential and the property of the University of Nottingham. No part of it may be transmitted, reproduced, published, or used by others persons without prior written authorisation from the University of Nottingham

Table 2 - Study Assessments

= RCT   = observational period

Outcomes collected	0 months	2 months	4 months	6 months	8 months
Informed consent for main study	√				
Informed consent for genetic study (optional)	√				
Eligibility checks	√				
Eczema severity (NESS)	√				
Supply garments	√ intervention			√ control	
Demographics	√				
Collect saliva sample (Optional)	√				
Issue diary	√	√	√	√	
EASI & TIS	√	√	√	√	
Investigator and Patient Global Assessment (IGA and PGA)	√	√	√	√	
Topical treatment usage	√	√		√	
Medication for skin infection	√	√	√	√	
Use of wet and dry wraps	√	√	√	√	
On-line questionnaire (weekly) including POEM, topical treatment use and use or wet and dry wraps		√	√	√	
Final on-line questionnaire					√
DFI	√			√	
EQ-5D-3L of parent	√			√	
Child utility scales (ADQoL, CHU-9D)	√			√	
Number of infections	√	√	√	√	
Serious adverse events		√	√	√	
NHS and family resource use	√	√ & Diary	√ & Diary	√ & Diary	√
Adherence		Ques	Ques	Ques	Ques
Durability of clothing				√	√
Acceptability (parent & child)				√	√
Replace garments if required (intervention group only)		√	√		

NESS = Nottingham Eczema Severity Scale; TIS = Three Item Severity scale; EASI – Eczema Area and Severity Index; POEM = Patient Oriented Eczema Measure; DFI = Dermatitis Family Impact questionnaire; EQ-5D-3L – EuroQol Five Dimension, Three Levels questionnaire; CHU-9D – The Child Health Utility 9 dimensions; Ques = Weekly on-line/postal quesionnaire

This protocol is confidential and the property of the University of Nottingham. No part of it may be transmitted, reproduced, published, or used by others persons without prior written authorisation from the University of Nottingham

Data Collection Methods

Face-face visits with the research nurse

These interviews will take place at baseline (recruitment visit), 2 months, 4 months and 6 months.

The visits will include the following:

i. check of eligibility criteria (recruitment visit only)
ii. baseline characteristics / demographics (recruitment visit only)
iii. collection of single saliva sample –optional (planned to be collected at the recruitment visit but can be collected at any visit according to patient preference)
iv. examining the child's skin for disease severity and signs of infection (using the Eczema Area and Severity Index,EASI); Three Item Severity score – TIS; and Investigator Global Assessment, IGA).
v. Patient-reported eczema severity (Patient Global Assessment. PGA) and number of skin infections treated with antibiotics or anti-viral treatments
vi. Review of topical medication usage (prescriptions issued and changes in treatment regimen)
vii. Review of health resource use (e.g. visits to GPs or nurses in relation to eczema treatment)
viii. Quality of life questionnaires (at baseline and 6 months only) – dermatitis family impact questionnaire, EQ-5D, eczema utility scale and CHU-9D.

Treatment diaries /memory aides
All participants will be given an eczema diary as an aide memoir to record health resource use and a sticker chart to record progress in the trial – participants randomised to wear the clothing for the first six months will have a chart where they can record when they have worn the clothing and participants randomised to the control group will have a chart to record each week they have been in the trial.
.
Each week the parent / legal guardian, if they have expressed a wish to complete the questions on-line will be sent a link (via e-mail) to an on-line questionnaire. This short questionnaire will be completed by referring to the diary / wall chart, and will record how their eczema has been that week, health service resource use (including, prescriptions and health professional contacts related to eczema), days of usage of: topical steroids, emollients, topical calcineurin inhibtors and wet/dry wraps, and adherence with / the clothing (intervention group only).

Participants who do not wish to complete the questions on-line will be asked to complete a form and post it back to the Clinical Trials Unit.

End of Trial Follow-up Questionnaire
Parents / legal guardians of participants will be sent an end of trial follow-up questionnaire (on-line or postal) at eight months post-randomisation. This will seek information about current eczema status, adherence, acceptability and their opinion of the therapeutic clothing.

Concomitant Treatment
Participants will be asked not to start new eczema treatments (other than antibiotics for infection) unless medically indicated, and will be discouraged from routinely using wet or dry wrap dressings. Any changes to a child's treatment regimen, or use of wet / dry wrapping will be recorded, but the child will remain in the trial.

Compliance

This protocol is confidential and the property of the University of Nottingham. No part of it may be transmitted, reproduced, published, or used by others persons without prior written authorisation from the University of Nottingham

Compliance with the protocol will be monitored. This will include following up patients who have not completed questionnaires and/or attended for clinic visits. Any instances of the research nurse being unblinded will be recorded. Adherence in wearing the garments will be collected weekly until 6 months and then at the end of the study (8 months). Participants randomised to wear the clothing for the first six months will have the option of using a sticker or tick chart to record the days and nights when they wear the clothing, which will act as an aid when they complete the questionnaires.

Criteria for terminating trial

This trial involves CE marked clothing that are being used within their normal licensing authorisation. As such, it is very low risk. However, if severe adverse effects associated with the clothing occur frequently, the study may be terminated on the advice of the Trial Steering Committee.

The Investigator may stop the trial or terminate one centre if new information becomes available causing major safety concerns, or if there are issues with trial conduct.

Should the trial be terminated the research data will not be destroyed.

OPTIONAL QUALITATIVE INTERVIEW/ FOCUS GROUP

For the sub-group who choose to participate in the qualitative study:

Data will be collected through

- 2 x child focus groups (5-8 years) n=16
- 4 x parent / guardian focus groups n=32
- Up to 10 x parent / guardian interviews, telephone or face-to-face *(only if insufficient recruitment to focus groups)*
- Up to 10 x child interview (>8 years) face-to-face (with parent / guardian present) *(only if many older children recruited to the main trial)*
- 20 x telephone interviews with prescribers and commissioners

<u>Parent / guardian Focus Groups</u>

Qualitative data will be collected from up to four audio-taped focus groups each of which will have up to eight participants; two of these groups will be run concurrently with the child focus groups. We will examine parent / guardian's experience of silk garment use and the impact this has had on the child and family.

The Investigator moderating the focus group will:

- Guide and encourage each parent / guardian to share their knowledge and experience
- Listen effectively and learn from the group
- Capture the essence of the discussion

Focus groups will be planned to ensure that they are convenient for parent / guardians; they will last for up to 2.5 hours. Each group will begin with

- Introductions
- Clarification of the purpose of the study
- Explanation of confidentiality (highlighting that whilst researchers can assure confidentiality absolute confidentiality from other members of the group cannot be guaranteed by the researcher).
- Reiteration of what is expected
- Reconfirmation that the focus group will be audio taped

This protocol is confidential and the property of the University of Nottingham. No part of it may be transmitted, reproduced, published, or used by others persons without prior written authorisation from the University of Nottingham

- Signing of consent forms

Questions posed to the groups will be open and use straightforward conversational language, the topic guide may include

- Grand tour question
  - Tell me a bit about the eczema, what it's like living with it
- Mini tour questions
  - How have you got on with the special clothing?
  - How much did you / your child wear the clothing? (day / night / away from home)
  - What was it like wearing the clothing? (skin condition, comments from others)
  - How did you get on with looking after the garments (washing, size)
- Example questions
  - Can you tell me about any differences you have noticed? (skin condition/ behaviour / wellbeing)
- Experience questions
  - Were there particular things you or your child liked or did not like about using the special garments?
  - How would you feel about continuing to use the special clothing (what things might make you continue or make you stop, barriers to use)?
- Supplementary question
  - How did you find completing the online questionnaires and paper diaries?
  - Do you have any preferences about how this information is collected?

Parent / guardian interviews

In the event of inadequate recruitment to the parent / guardian focus groups we will invite parents / guardians to take part in individual, audio recorded, telephone or face-to-face interviews lasting up to 1 hour using the topic guide outlined above.

Child Focus Groups

To ensure that focus groups are age appropriate the children will be divided into two age brackets: 5 – 6 years; 7 – 8 years with a group for each age range each with a maximum of eight children (total n=16). The children's groups will last for a maximum of 2.5 hours. Parent / guardians will meet facilitators and be invited to ask any questions they may have at the beginning of their child's focus group. Groups will be moderated by an experienced children's practitioner assisted by a note-taking observer.

The activities used will depend on the individual participating children's needs, wishes and level of understanding. Time will be allowed for the children to become familiar with the environment, facilitators and other children. This time will enable the facilitators to assess individual and group needs in terms of activities that they enjoy.

Each group will be introduced using a 'circle time' approach with which most children are likely to be familiar The facilitator (EW) will work with the children to build a story about 24 hours in the life of a child who has eczema and who has been wearing silk clothing. A story sack will be used which will contain different items according to the age and developmental ability of the children. Semi-structured play will be encouraged using various props that reflect the individual choices of the children and thus enables the children to express their thoughts in a way that is meaningful to them rather than make adult focused assumptions. Props will include for example:

- A doll wearing silk clothing
- Playmobile
- Colouring activities

This protocol is confidential and the property of the University of Nottingham. No part of it may be transmitted, reproduced, published, or used by others persons without prior written authorisation from the University of Nottingham

- Stickers
- Puppets
- Talking tins
- Foam people of varying sizes for the children to decorate as part of the story as it evolves
- Foam faces
- Role play props to help the children visualise the activities during the day within the story they are developing

EW will lead the story telling and activities and the observer will gather data by writing down key quotes from children and by photographing any artefacts produced and recording, in writing, children's explanations of what they have made. At the end of the story that the children have made, a recap of the event will take place with the children and each aspect of the story shared with them to ensure that the final story reflects their thoughts and feelings. Digital photographs of the artefacts (not the children) will be recorded and we will offer to email the photographs to the children.

Child interviews

Children aged >8 will be invited to take part in individual, audio recorded face-to-face interviews lasting up to 1 hour. Depending on the age or interests of the child, they will either express their views through similar hands-on activities proposed to the younger children, or invited to have a one-on-one informal chat. They will be accompanied by a parent / guardian at all times.

Topic guide may include

- Grand tour question
  - Tell me a bit about your eczema, what it's like living with it
- Mini tour questions
  - How have you got on with the special clothing?
  - How much did you wear the clothing? (day / night / away from home)
  - What was it like wearing the clothing? (skin condition, comments from others)
- Example questions
  - Can you tell me about any differences you have noticed? (skin condition / wellbeing)
- Experience questions
  - Were there particular things you liked or did not like about using the special garments?

All digital recordings will be transcribed verbatim and then deleted from the recording device. Data including transcripts and artefacts from the child focus groups will be anonymised and stored securely on password protected computers.

Prescriber and commissioner interviews

Telephone interviews lasting 20-30 minutes will be planned both theoretically and technically. The interview schedule will be pre-tested and will be sent to participants prior to the interview and a convenient time for the call will be arranged. Interviews will, with permission be audio-taped and written notes recorded. In some instances participants may be both prescribers and commissioners in this case questions will be adjusted accordingly.

- What is your experience of prescribing silk garments for children with eczema?
- What makes you select this line of treatment?

This protocol is confidential and the property of the University of Nottingham. No part of it may be transmitted, reproduced, published, or used by others persons without prior written authorisation from the University of Nottingham

- Are there any barriers to you enabling children to use these garments?
    - Perceived cost
    - Lack of evidence
    - Durability of the garments
- Do you think it is a reasonable expectation that GPs should prescribe silk garments?
- If no, who do you think is the most appropriate person to prescribe these garments?
- Do you think they should be prescribed at all? Can you explain the reason for this?

All digital recordings will be transcribed verbatim and then deleted from the recording device. Data including transcripts and artefacts from the child focus groups will be anonymised and stored securely on password protected computers.

FILLAGGRIN GENETICS ANALYSES

Saliva testing for mutations on the gene encoding for filaggrin (FLG)
A sub-group analysis based on presence or absence of mutation(s) in the FLG will be conducted. Mutations in FLG are commonly found in eczema patients (25 -50% depending on the population(Thomas, Armstrong et al. 2002), and has been associated with disruption of skin barrier function and dry skin((Palmer, Irvine et al. 2006). As a result, there is a strong theoretical basis for thinking that children with at least one FLG mutation would be more likely to benefit from clothing, which potentially acts to improve the barrier function of the skin.

In light of this we plan to include FLG genotype as a possible predictor of treatment response in the current study.

For the sub-group analysis, study participants will be tested for up to six prevalent FLG loss-of-function mutations, depending on quality/quantity of DNA (R501X, 2282del4, R2447X ,S3247X, 3702delG and 3673delC) and categorised into two groups according to FLG status:
Group 1: FLG +/+ (no mutations) – control cohort
Group 2: FLG +/- (carrying one FLG null mutation) and FLG -/- (carrying two FLG null mutations)

This work will refine the phenotypic characteristics of filaggrin-deficient eczema, and will assess whether FLG genotype is an important predictive factor in determining treatment success.

Methodology
To determine FLG genotype it will be necessary to obtain DNA from individuals entering the study. Following additional written informed consent, participants will be asked to provide a saliva sample at enrolment (or at their subsequent appointments if more practical). If children are unable to spit into the container, swabs taken from inside the cheek will be used to collect the sample.

Sample containers will be identified using the designated study number and date of birth only. Personal contact details will be kept by the Trial team and will not be transferred to the laboratory researchers.

The containers will be shipped to Dr Sara Brown at the Centre for Dermatology & Genetic Medicine, University of Dundee. DNA will be extracted by standard techniques and FLG genotyping for the four most prevalent null-alleles (R501X, 2282del4, R2447X and S3247X)

This protocol is confidential and the property of the University of Nottingham. No part of it may be transmitted, reproduced, published, or used by others persons without prior written authorisation from the University of Nottingham

and depending on the quality and quantity of DNA in each individual sample, two more mutations will also be tested for (3702delG and 3673delC). All DNA testing will be carried out according to published protocols (Sandilands, Terron-Kwiatkowski et al. 2007). Samples will be securely stored within the University of Dundee for future testing of genes associated with atopic eczema. If new techniques become available, consent will be obtained for this.

FLG genotype status will be recorded and returned to the Nottingham Clinical Trials Unit again using the study number and date of birth.

STATISTICS

Methods

All analyses will be carried out in accordance with CONSORT guidelines using Stata/SE 11.2 or MLwiN v2.2. Appropriate descriptive statistics will be used to compare randomised participants with those who were eligible and not randomised, and to compare the randomised arms at baseline. The primary approach to analysis will be intention-to-treat (ITT) without imputation. The primary analysis of EASI will be performed using a multilevel model (MLM) framework, with observations at 2, 4 and 6 months (level 1) nested within participants (level 2) and including baseline EASI and the stratification factors (site and age group) as covariates

Supportive analyses of the primary end point, analyses of secondary and safety end points, planned sub-group analyses, sensitivity analyses including imputation of missing outcome data and accounting for varying adherence with allocation, and any further exploratory analyses will be conducted using appropriate multivariable regression models and documented in the Statistical Analysis Plan (SAP) which will be finalised prior to unblinding of the study. Any changes in the planned statistical methods will be documented in the trial report.

Sample size and justification

Three hundred participants provides 90% power, at the 5% significance level (two-tailed) to detect a difference of around 3 points between the groups in mean EASI scores over 2, 4 and 6 months using a repeated measures analysis of covariance, assuming a SD of 13, a correlation between EASI scores at different time points of 0.6 and loss to follow up of 10%.

A 3-point improvement in EASI represents a small but still clinically meaningful difference between groups, and we are keen to ensure that the study is sufficiently powered to detect this magnitude of difference since it is unlikely that a trial like this will be done again. A small treatment response could still be worthwhile to the NHS since this non-pharmacological treatment is assume to have no adverse effects, and because eczema affects so many people. It is also likely that a relatively small response on the objective primary outcome will be reflected in larger, more clinically meaningful treatment effects in the patient-reported outcomes.

Participants will be recruited from six recruiting centres over an 18 month recruitment period.. Recruitment is expected to increase gradually over the first 6 months of the trial to allow for staggered site set-up.

Procedures for missing, unused and spurious data

Every effort will be made within the trial to minimise the occurrence of missing data. All missing data items will be tabulated by treatment arm and reasons given where possible. We will examine the plausibility that data are missing at random (MAR) and multiple imputation techniques will be used to handle missing values as appropriate.

This protocol is confidential and the property of the University of Nottingham. No part of it may be transmitted, reproduced, published, or used by others persons without prior written authorisation from the University of Nottingham

Data from the parent / guardian and child focus groups and interviews and prescriber and commissioner interviews will be analysed separately using a manual process of thematic analysis. Audio tapes will be transcribed verbatim. Transcripts, notes and artefacts (as explained by children) will be reviewed in detail, coding the substance of the data. Theoretical codes will be added beside each code as a reminder of any thoughts or questions attached to the codes. As codes develop they will be compared with the aim of identifying similarities and differences amongst incidence in the data. The identified codes will be condensed into higher levels of abstraction to form categories; these will be continuously reviewed to ensure that they cover variations within the data. The final step will be to interpret the categories so that better understanding is achieved. To ensure trustworthiness members of the research team will be involved in the analysis process.

Data from this qualitative study will be linked with data from the RCT to provide a rounded assessment of the impact of wearing silk garments and the patient-reported factors that may influence this.

This protocol is confidential and the property of the University of Nottingham. No part of it may be transmitted, reproduced, published, or used by others persons without prior written authorisation from the University of Nottingham

COST-EFFECTIVENESS ANALYSIS

Objective:

To estimate the within trial cost-effectiveness of silk therapeutic clothing with standard care compared to standard care alone from an NHS and a family perspective.

The cost analysis will compare the overall costs for the intervention to standard care, measuring resource use such as primary care contacts, medication prescribed, secondary care contacts and patient costs. Health and family resource use data will be recorded by research nurses. The patients will be asked to collect data on a diary which will serve as an aid memoire.. Resource use will be valued using published unit costs (e.g. Curtis and Netten (PSSRU 2004), BNF 2005, and NHS reference costs and patient reported estimates. The costs to the NHS and patient will be reported separately as well as in combination.

The primary measures of effectiveness for the cost effectiveness analysis will be the difference in number of participants who achieved "treatment success" (defined as those with at least a 50% improvement in EASI at 6 months compared to baseline). Secondary analyses will be conducted using continuous data from the EASI scale; the Dermatitis Family Impact Scale; eczema specific utility measures (Stevens 2005) and generic measures of health utility as measured using the EQ-5D-3L (for the main carer) and the Children's Health Utility Index 9D (CHU-9D) for children. A cost utility analysis where effectiveness is measured in terms of the Quality Adjusted Life Years (QALYs) for child and main carer will be undertaken (using linear interpolation and area under the curve with baseline adjustment).

If non-dominance occurs an incremental cost-effectiveness ratio will be produced. Sensitivity analysis will be undertaken to test the robustness of results in the face of any uncertainties or assumptions made in the analysis.

ADVERSE EVENTS

It is unlikely that silk clothing will result in any adverse effects. As such AEs will not be collected for this trial, but ALL SAEs will be.

A worsening of eczema and infected eczema will not be considered as adverse events as these data will be collected as specific endpoints for the trial. A worsening of eczema will be detected using the scores from EASI.

Hospitalisation due to eczema will be captured as a Serious Adverse Event (SAE).

Trial Intervention-related SAEs

A serious adverse event that is unexpected in its severity and seriousness *and* deemed **directly related to or suspected to be related to the trial intervention** shall be reported to the ethics committee that gave a favourable opinion as stated below.

The event shall be reported immediately of knowledge of its occurrence to the Chief Investigator, and to the clinician named responsible for evaluating SAEs on the trial delegation log

The site will:

- Report all SAEs to the trial coordinating unit (Nottingham Clinical Trials Unit) via fax, using the University of Nottingham form (TA014).

The Trial Coordinating Unit will:

This protocol is confidential and the property of the University of Nottingham. No part of it may be transmitted, reproduced, published, or used by others persons without prior written authorisation from the University of Nottingham

- Send SAEs to the CI and clinician delegated to evaluate SAEs.

The Clinician named responsible for evaluating SAEs will:

- Assess the event for seriousness, expectedness and relatedness to the trial treatment or intervention.
- Take appropriate medical action, which may include halting the trial and inform the Sponsor of such action.
- If the event is deemed related to the trial treatment or intervention shall inform the REC using the reporting form found on the NRES web page within 7 days of knowledge of the event.
- Shall, within a further eight days send any follow-up information and reports to the REC.
- Make any amendments as required to the study protocol and inform the REC as required

ETHICAL AND REGULATORY ASPECTS

ETHICS COMMITTEE AND REGULATORY APPROVALS

The trial will not be initiated before the protocol, informed consent forms and participant and GP information sheets have received approval / favourable opinion from the Research Ethics Committee (REC), and the respective National Health Service (NHS) Research & Development (R&D) department. Should a protocol amendment be made that requires REC approval, the changes in the protocol will not be instituted until the amendment and revised informed consent forms and participant and GP information sheets (if appropriate) have been reviewed and received approval / favourable opinion from the REC and R&D departments. A protocol amendment intended to eliminate an apparent immediate hazard to participants may be implemented immediately providing that the REC are notified as soon as possible and an approval is requested. Minor protocol amendments only for logistical or administrative changes may be implemented immediately; and the REC will be informed.

The trial will be conducted in accordance with the ethical principles that have their origin in the Declaration of Helsinki, 1996; the principles of Good Clinical Practice, and the Department of Health Research Governance Framework for Health and Social care, 2005.

This protocol is confidential and the property of the University of Nottingham. No part of it may be transmitted, reproduced, published, or used by others persons without prior written authorisation from the University of Nottingham

Specific issues for the qualitative element are summarised below:

Safety: qualitative data will be collected using focus groups and individual interviews. Parent / guardians and children will participate in separate groups although they will be held concurrently in one large room in a local amenity. This will allow parent / guardians to maintain visual contact with their children at all times. Focus groups will be conducted at weekends or during school holidays to ensure that the children are not overtired. The Investigators who will conduct the child focus groups have considerable experience in interacting with young children and have enhanced CRB checks. They will be conversant with the current national guidance in relation to safeguarding children (Department of Education, 2013). If children do not wish to participate in the focus groups they will be able to return to their parent / guardian with ease.

Rights: Participant information sheets will be provided for parent / guardians and children and they will have the opportunity to discuss the study before agreeing to take part. Participants will be identified and contacted by the research team in the first instance. If they wish to take part they will be asked to contact the CI (FC) and provide contact details. Any contact details will be stored securely and separately from other data. Other data will be anonymised.

Wellbeing: the researcher will ensure that participants are fully briefed about the study. The Investigator who will conduct the child focus groups is conversant with the current safeguarding children national guidance and will follow the safeguarding children policies and procedures for the locality where the research is being undertaken should any concerns regarding a child's wellbeing be raised. Participants will be informed that information from focus group interviews will only be used for the study. The only exception is that if any information disclosed to Investigators may be of concern in relation to safeguarding (child protection) issues this will be reported to the child's health care team.

Dignity: the participants will be treated with dignity at all times during the study.

INFORMED CONSENT AND PARTICIPANT INFORMATION

The process for obtaining assent and parent / guardian informed consent will be in accordance with the REC guidance, and Good Clinical Practice (GCP) and any other regulatory requirements that might be introduced. The investigator or their nominee and the participant or other legally authorised representative shall both sign and date the Informed Consent Form before the person can participate in the study. Children who wish to do so, will also be able to provide "assent" to participation in the trial on the same form.

The participant (or parent/guardian) will receive a copy of the signed and dated forms and the original will be retained in the Trial Master File. A second copy will be filed in the participant's medical notes and a signed and dated note made in the notes that informed consent was obtained for the trial.

The decision regarding participation in the study is entirely voluntary. The investigator or their nominee shall emphasize to them that consent regarding study participation may be withdrawn at any time without penalty or affecting the quality or quantity of their future medical care, or loss of benefits to which the participant is otherwise entitled. No trial-specific interventions will be done before informed consent has been obtained.

The investigator will inform the participant (where appropriate) and their parent/guardian of any relevant information that becomes available during the course of the study, and will discuss with them, whether they wish to continue with the study. If applicable they will be asked to sign revised consent forms.

This protocol is confidential and the property of the University of Nottingham. No part of it may be transmitted, reproduced, published, or used by others persons without prior written authorisation from the University of Nottingham

If the Informed Consent Form is amended during the study, the investigator shall follow all applicable regulatory requirements pertaining to approval of the amended Informed Consent Form by the REC and use of the amended form (including for ongoing participants).

RECORDS

Case Report Forms and patient diaries

Each participant will be assigned a trial identity code number, allocated at randomisation if appropriate, for use on CRFs other trial documents and the electronic database. The documents and database will also use their initials (of first and last names separated by a hyphen or a middle name initial when available) and date of birth (dd/mmm/yyyy).

CRFs will be treated as confidential documents and held securely in accordance with regulations. The investigator will make a separate confidential record of the participant's name, date of birth, local hospital number or NHS number, and Participant Trial Number (the Trial Recruitment Log), to permit identification of all participants enrolled in the trial, in accordance with regulatory requirements and for follow-up as required
CRFs shall be restricted to those personnel approved by the Chief or local Principal Investigator and recorded on the 'Trial Delegation Log.'

All paper forms shall be completed and collected in line with GCP. The Chief or local Principal Investigator shall sign a declaration ensuring accuracy of data recorded in the CRF.

Sample Labelling

Each participant will be assigned a trial identity code number for use on the samples, consent forms and other study documents and the electronic database. The documents and database will also use their initials (of first and last names separated by a hyphen or a middle name initial when available) and date of birth (dd/mmm/yyyy).

Source documents

Source documents shall be filed at the investigator's site and may include but are not limited to, consent forms, current medical records and diaries. A CRF may also completely serve as its own source data. Only trial staff as listed on the Delegation Log shall have access to trial documentation other than the regulatory requirements listed below.

Direct access to source data / documents

The CRF and all source documents, including progress notes and copies of laboratory and medical test results shall made be available at all times for review by the Chief Investigator, Sponsor's designee and inspection by relevant regulatory authorities (e.g. DH, Human Tissue Authority).

DATA PROTECTION

All trial staff and investigators will endeavour to protect the rights of the trial's participants to privacy and informed consent, and will adhere to the Data Protection Act, 1998. The CRF will only collect the minimum required information for the purposes of the trial. CRFs will be held securely, in a locked room, or locked cupboard or cabinet. Access to the information will be limited to the trial staff and investigators and relevant regulatory authorities (see above). Computer held data including the trial database will be held securely and password

This protocol is confidential and the property of the University of Nottingham. No part of it may be transmitted, reproduced, published, or used by others persons without prior written authorisation from the University of Nottingham

protected. All data will be stored on a secure dedicated web server. Access will be restricted by user identifiers and passwords (encrypted using a one way encryption method). Information about the trial in the participant's medical records / hospital notes will be treated confidentially in the same way as all other confidential medical information.

Electronic data will be backed up every 24 hours to both local and remote media in encrypted format.

QUALITY ASSURANCE & AUDIT

INSURANCE AND INDEMNITY

Insurance and indemnity for trial participants and trial staff is covered within the NHS Indemnity Arrangements for clinical negligence claims in the NHS, issued under cover of HSG (96)48. There are no special compensation arrangements, but trial participants may have recourse through the NHS complaints procedures.

The University of Nottingham as research Sponsor indemnifies its staff, research participants and research protocols with both public liability insurance and clinical trials insurance. These policies include provision for indemnity in the event of a successful litigious claim for proven non-negligent harm.

TRIAL CONDUCT

Trial conduct will be subject to systems audit of the Trial Master File for inclusion of essential documents; permissions to conduct the trial; Trial Delegation Log; CVs of trial staff and training received; local document control procedures; consent procedures and recruitment logs; adherence to procedures defined in the protocol (e.g. inclusion / exclusion criteria, correct randomisation, timeliness of visits); adverse event recording and reporting; accountability of trial materials and equipment calibration logs.

TRIAL DATA

Monitoring of trial data shall include confirmation of informed consent; source data verification; data storage and data transfer procedures; local quality control checks and procedures, back-up and disaster recovery of any local databases and validation of data manipulation. The Trial Coordinator, or where required, a nominated designee of the Sponsor, shall carry out monitoring of trial data as an ongoing activity.

Entries on CRFs will be verified by inspection against the source data. A sample of CRFs will be checked on a regular basis for verification of all entries made. In addition the subsequent capture of the data on the trial database will be checked. Where corrections are required these will carry a full audit trail and justification.

Trial data and evidence of monitoring and systems audits will be made available for inspection by REC as required.

RECORD RETENTION AND ARCHIVING

In compliance with the ICH/GCP guidelines, regulations and in accordance with the University of Nottingham Research Code of Conduct and Research Ethics, the Chief or local Principal Investigator will maintain all records and documents regarding the conduct of the study. These will be retained for at least 7 years or for longer if required. If the responsible investigator is no longer able to maintain the study records, a second person will be nominated to take over this responsibility.

This protocol is confidential and the property of the University of Nottingham. No part of it may be transmitted, reproduced, published, or used by others persons without prior written authorisation from the University of Nottingham

The Trial Master File and trial documents held by the Chief Investigator on behalf of the Sponsor shall be finally archived at secure archive facilities at the University of Nottingham. This archive shall include all trial databases and associated meta-data encryption codes.

DISCONTINUATION OF THE TRIAL BY THE SPONSOR

The Sponsor reserves the right to discontinue this trial at any time for failure to meet expected enrolment goals, for safety or any other administrative reasons. The Sponsor shall take advice from the Trial Steering Committee as appropriate in making this decision.

STATEMENT OF CONFIDENTIALITY

Individual participant medical information obtained as a result of this study are considered confidential and disclosure to third parties is prohibited with the exceptions noted above.
Participant confidentiality will be further ensured by utilising identification code numbers to correspond to treatment data in the computer files.

Such medical information may be given to the participant's medical team and all appropriate medical personnel responsible for the participant's welfare.

Data generated as a result of this trial will be available for inspection on request by the participating physicians, the University of Nottingham representatives, the REC, local R&D Departments and the regulatory authorities.

PUBLICATION AND DISSEMINATION POLICY

During the period of the trial, press releases will be issued from the Centre of Evidence Based Dermatology. No party will be entitled to submit any publicity material without prior approval from the co-ordinating centre.
Trial publications and conference presentations will be submitted to the HTA for approval prior to submission to the event organisers or the editors. All publications will acknowledge the support of the HTA in funding this trial.
All participants will receive a copy of the trial results in the form of a participant newsletter.
Neutral or negative results will not constitute a reasonable justification to delay publication

USER AND PUBLIC INVOLVEMENT

The public and patients have been involved in the prioritising of this research questions as outline in the Eczema Priority Setting Partnership(Batchelor, Ridd et al. 2013). In addition there has been public involvement in the trial design, the patient information, on-line questionnaires and diary cards. There will be at least one member of the public with an interest in eczema on the trial steering group

STUDY FINANCES

Funding source

This study is funded by NIHR Health Technology Assessment - Ref 11/65/01

Participant stipends and payments

Participants will not be paid to participate in the trial. Travel expenses will be offered for any hospital visits in excess of usual care and small gifts, sticker incentives will be given to children in order to encourage continued participation and completion of study diaries.

This protocol is confidential and the property of the University of Nottingham. No part of it may be transmitted, reproduced, published, or used by others persons without prior written authorisation from the University of Nottingham

SIGNATURE PAGES

Signatories to Protocol:

Chief Investigator: (name) Kim Thomas

Signature: K S Thomas

Date: 12/2/2014

Trial Statistician: (name) Alan Montgomery

Signature: A. Montgomery

Date: 12/2/2014.

This protocol is confidential and the property of the University of Nottingham. No part of it may be transmitted, reproduced, published, or used by others persons without prior written authorisation from the University of Nottingham

REFERENCES

Barbier, N., C. Paul, et al. (2004). "Validation of the Eczema Area and Severity Index for atopic dermatitis in a cohort of 1550 patients from the pimecrolimus cream 1% randomized controlled clinical trials programme." Br J Dermatol **150**(1): 96-102.

Batchelor, J. M., M. J. Ridd, et al. (2013). "The Eczema Priority Setting Partnership: a collaboration between patients, carers, clinicians and researchers to identify and prioritize important research questions for the treatment of eczema." Br J Dermatol **168**(3): 577-582.

Charman, C., A. Venn, et al. (2004). "The Patient-Oriented Eczema Measure: Development and Initial Validation of a New Tool for Measuring Atopic Eczema Severity From the Patients' Perspective." Arch Dermatol **140**(12): 1513-1519.

Diepgen, T., B. Salzer, et al. (1995). "A study of skin irritations by textiles under standardised sweating conditions in patients with atopic eczema." Melliand English **12**.

Emerson, R. M., Charman, C.R., Williams, H.C. (2000). "The Nottingham Eczema Severity Score: preliminary refinement of the Rajka and Langeland grading." British Journal of Dermatology **142**: 288-297.

Fabsitz, R. R., A. McGuire, et al. (2010). "Ethical and practical guidelines for reporting genetic research results to study participants: updated guidelines from a National Heart, Lung, and Blood Institute working group." Circ Cardiovasc Genet **3**(6): 574-580.

Gauger, A., S. Fischer, et al. (2006). "Efficacy and functionality of silver-coated textiles in patients with atopic eczema." J Eur Acad Dermatol Venereol **20**(5): 534-541.

Hoare, C., A. Li Wan Po, et al. (2000). "Systematic review of treatments for atopic eczema." Health Technol Assess **4**(37): 1-191.

Juenger, M., A. Ladwig, et al. (2006). "Efficacy and safety of silver textile in the treatment of atopic dermatitis (AD)." Curr Med Res Opin **22**(4): 739-750.

Koller, D. Y., G. Halmerbauer, et al. (2007). "Action of a silk fabric treated with AEGIS in children with atopic dermatitis: a 3-month trial." Pediatr Allergy Immunol **18**(4): 335-338.

Lawson, V., S. Lewis-Jones, et al. (1998). "The family impact of childhood atopic dermatitis: the dermatitis family impact questionnaire." Br J Dermatol **138**: 107-113.

NICE (December 2007). NICE clinical guideline 57: Management of atopic eczema in children from birth up to the age of 12 years, Department of Health.

Ozawa, M. (2008). "Effect of underwear made from MEDIELE on skin barrier function of atopic dermatitis patients in winter season." Skin Research **7**(4): 7.

Palmer, C. N., A. D. Irvine, et al. (2006). "Common loss-of-function variants of the epidermal barrier protein filaggrin are a major predisposing factor for atopic dermatitis." Nat Genet **38**(4): 441-446.

PSSRU (2004). Unit costs of health and social care. Canterbury, Personal social services research unit.

Sandilands, A., A. Terron-Kwiatkowski, et al. (2007). "Comprehensive analysis of the gene encoding filaggrin uncovers prevalent and rare mutations in ichthyosis vulgaris and atopic eczema." Nat Genet **39**(5): 650-654.

Stevens, K. (2012). "Valuation of the Child Health Utility 9D Index." Pharmacoeconomics **30**(8): 729-747.

Stevens, K. J., J. E. Brazier, et al. (2005). "The development of a preference-based measure of health in children with atopic dermatitis." Br J Dermatol **153**(2): 372-377.

Stinco, G., F. Piccirillo, et al. (2008). "A randomized double-blind study to investigate the clinical efficacy of adding a non-migrating antimicrobial to a special silk fabric in the treatment of atopic dermatitis." Dermatology **217**(3): 191-195.

Thomas, K. S., S. Armstrong, et al. (2002). "Randomised controlled trial of short bursts of a potent topical corticosteroid versus prolonged use of a mild preparation for children with mild or moderate atopic eczema." BMJ **324**(7340): 768.

Vlachou, C., K. S. Thomas, et al. (2009). "A case report and critical appraisal of the literature on the use of DermaSilk in children with atopic dermatitis." Clin Exp Dermatol **34**(8): e901-903.

Williams, H., P. Burney, et al. (1994). "The UK working party's diagnostic criteria for atopic dermatitis. I. Derivation of a minimum set of discriminators for atopic dermatitis." Br J Dermatol **131**(3): 383-396.

Williams, H., A. Stewart, et al. (2008). "Is eczema really on the increase worldwide?" J Allergy Clin Immunol **121**(4): 947-954 e915.

Yokoyama, Y., H. Kimata, et al. (2009). "Ethylene vinyl alcohol (EVOH) fiber compared to cotton underwear in the treatment of childhood atopic dermatitis: a double-blind randomized study." Indian Pediatr **46**(7): 611-614.

This protocol is confidential and the property of the University of Nottingham. No part of it may be transmitted, reproduced, published, or used by others persons without prior written authorisation from the University of Nottingham
